# A Simple HPLC-DAD Method for the Therapeutic Monitoring of Clozapine and Related Metabolites in Human Plasma and Urine Samples

**DOI:** 10.3390/molecules29215039

**Published:** 2024-10-25

**Authors:** Mircea-Alexandru Comănescu, Dana-Maria Preda, Dalia-Simona Miron, Flavian-Ștefan Rădulescu, Victor Voicu, Andrei-Valentin Medvedovici

**Affiliations:** 1 Department of Analytical & Physical Chemistry, Faculty of Chemistry, University of Bucharest, Panduri Ave. # 90-92, 050663 Bucharest, Romania; 2 Center for Drug Sciences, Faculty of Pharmacy, University of Medicine and Pharmacy Carol Davila Bucharest, #6 Traian Vuia Street, 025956 Bucharest, Romania; 3 Section of Medical Science, Romanian Academy, Calea Victoriei # 125, 010071 Bucharest, Romania

**Keywords:** clozapine, metabolites, HPLC-DAD, LLE, *n*-octanol, SUPRAS

## Abstract

Clozapine and its metabolites require close therapeutic monitoring (TDM) in patients due to poor correlation between the administrated doses and resulting plasma concentrations, the narrow therapeutic interval, high inter-individual variability, and the risk of serious side effects once toxic levels are exceeded. The aim of the study was to develop a simple (relatively cheap) LC-UV method for the quantification of clozapine and its metabolites in plasma and urine samples. For sample preparation, liquid-liquid extraction (LLE) in *n*-octanol was more efficient and less limiting in injection volumes compared to the in-situ formation of SUPRAS. When analyzing urine, an alkalinization step before extraction was required. The proposed method produced linear concentration responses with/without internal standard (IS) for the target analytes, with LLOQs within the targeted range of 50 ppb and %RSD within the acceptable 15% range. Furthermore, sample stability studies proved that pre-extracted samples were stable for the short term at room temperature and long-term when frozen.

## 1. Introduction

Clozapine (CLO) is an antipsychotic drug primarily prescribed in cases of treatment-resistant schizophrenia; despite its usefulness, treatment plans using clozapine require monitoring due to clozapine’s potential for multiple adverse side effects on the heart (cardiomyopathy and myocarditis), gastrointestinal system (decreasing or stoppage of intestinal contents), and its potential for seizures and death [1,2,3]. Studies have also linked clozapine treatment complications when administered to smokers, or when co-prescribed with other drugs that have a potential for inhibiting CYP1A2 enzymatic activities and thus affecting clozapine’s metabolism, such as psychotropic (e.g., fluvoxamine) and non-psychotropic (e.g., contraceptives) medication [1,2,3,4].

**Figure 1:**
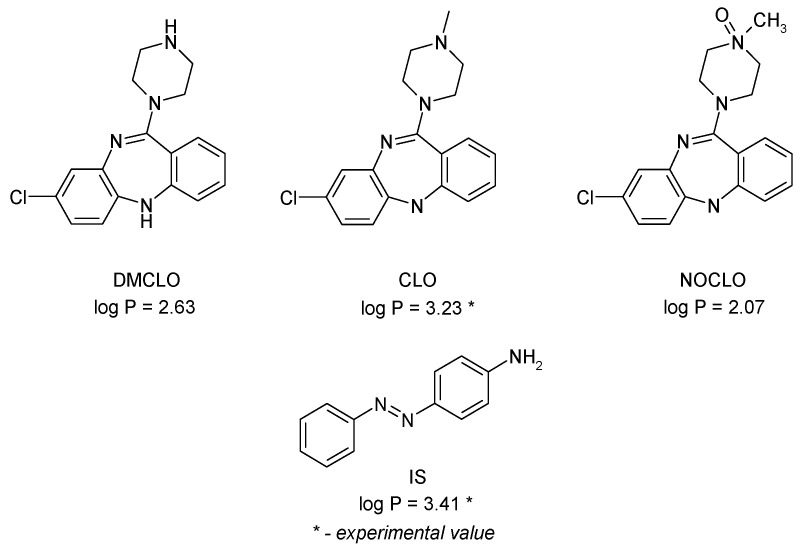
The molecular structures of the target compounds: Clozapine (CLO), metabolites Desmethylclozapine (DMCLO) and Clozapine N-Oxide (NOCLO), and Internal Standard (IS—Aniline Yellow). The experimental/calculated (fragment theory) values of log P are also given.

Clozapine is primarily metabolized to N-desmethylclozapine (DMCLO or norclozapine) by demethylation and clozapine N-oxide (NOCLO) by oxidation [4]. The molecular structures of the target analytes and internal standard (IS), together with the experimental log P values (denoted with *) or computed ones (KOWINT fragment theory from the EPI suite™ Estimation program interface from US EPA), are provided in Figure 1.

Monitoring of clozapine and/or its metabolites has been approached through various means, depending on the biological matrix that was to be analyzed. Serum samples have been evaluated through gas chromatography with traditional nitrogen-phosphorous or electron capture detectors [5,6] or mass spectrometry (MS) [7] detection, or through liquid chromatography with UV [8] or tandem MS [9] detection. To isolate the target analytes, serum samples have been processed via liquid-liquid extractions [5,6,9] or through commercial [7,8] or molecularly imprinted polymers [10] solid phase extraction columns. For plasma samples, the predominant instrumental method was liquid chromatography with UV detection [8,11,12,13,14,15,16,17,18,19,20,21,22,23] with either LLE or SPE methods, followed by LC-MS [24], LC-MS/MS [25], GC-MS [7], or capillary electrophoresis [26]. Whole blood samples were also approached from a GC-MS [7] or HPLC-UV [27] analysis perspective. Urine samples were analyzed by HPLC-UV [23] or LC-MS [28]. Other monitoring methods included analysis of dried blood spot samples via LC-MS/MS [29,30], wireless monitoring of clozapine concentrations in saliva via screen-printed electrochemical sensors [31], or post-mortem analysis of clozapine in the liver by potentiometric sensors [32].

In most studies [1], clozapine is reported to have a therapeutic blood concentration of at least 350 ng/mL, with patient samples showing 12-h post-dose concentrations of NOCLO between 0–34 ng/mL and combined DMCLO levels between 16–32 ng/mL. Urine levels [20] are reported as 1:43:77 (CLO:DMCLO:NOCLO) with 0.12% unchanged clozapine renal clearance of the administered dose. Certain aspects of these studies highlight that LLEs preferentially use an increased sample pH [5,9,11,24] and either performing back-extractions [11,16,22,28] or drying and reconstituting the extract [5,9,19,26] prior to analysis. Additionally, NOCLO was found to pose some separation issues from the CLO peak [5] or, alternatively, to have a stability issue whereby it converted back to CLO [27,29] and thus decreased the quantitative accuracy of analyses.

In this study, we propose a reversed-phase HPLC-DAD method for the analysis of CLO, DMCLO, and NOCLO from human plasma and urine, with sample processing via LLE, allowing quantitative determination of the target analytes within the therapeutic range. 

## 2. Results

### 2.1. Chromatographic Separation

**Figure 2:**
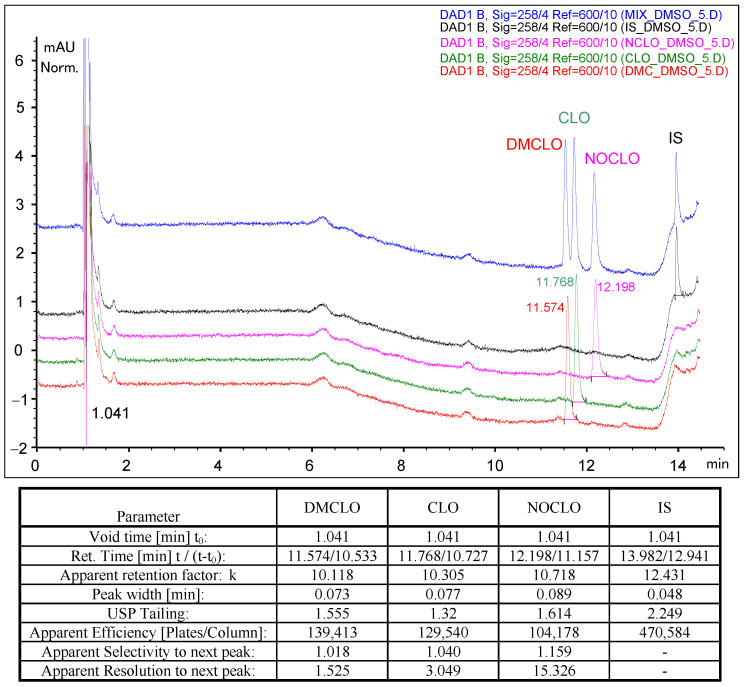
Separation of the target analytes (1 ppm in DMSO concentrations of each analyte, 5 μL injection, separation conditions as described in Section 4). Peak parameters are included in the figure.

Figure 2 shows the chromatographic separation of the target analytes according to the conditions described in Section 4. One can observe that the elution gradient profile generates apparent retention factors higher than 5 (frequently considered as a basic recommendation for bioanalytical approaches) and acceptable symmetry factors (<2). The apparent chromatographic resolution computed between each successive eluting pair of compounds can also be considered fair.

### 2.2. Instrumental Detection Limits

Given the quantitative aspect of this study, it was obviously necessary to evaluate the instrumental detection limits of the UV detection system. In Supplementary Materials, Figure S1, we present the calibration curves for the target compounds prepared in DMSO. The response function characteristics seen in Figure S1 are listed in Table 1 below. One can observe that the LLOQ limits are roughly 2–4 ng of the analytes loaded onto the column or the equivalent of 1 μL injected volumes of solutions with 2–4 μg/mL concentrations. This LLOQ makes it clear that in order to see targeted LLOQ limits of 50 ng/mL from biological samples, the sample preparation approaches and injection volumes should be modified to allow concentration of the sample by a factor of at least 2 (for the preparation stage) and at least 50 (for injection volumes). It should also be stated that from a statistical approach, using a weighted (1/x^2^) linear regression model is not, in this case, advantageous to determine LLOQ values.

### 2.3. Sample Preparation

#### 2.3.1. Liquid-Liquid Extraction in *n*-Octanol

Two different approaches were considered for the sample preparation stage. The first alternative was LLE extraction in *n*-octanol (log P of *n*-octanol, according to Hansch et al. [33], is 3, in accordance with the log P values of the analytes). The calibration curves for the target compounds dissolved in neat *n*-octanol when injecting a 50 μL volume are illustrated in the Supplementary Materials Section, Figure S2. The characteristics of the response functions produced for these injections are presented in Table 2. Three particular points should be made: first, the targeted LLOQ level of 50 ng/mL was reached; secondly, the weighted linear regression model has a worse performance on quantification limits; third, the use of the peak area ratio between analyte and IS as the dependent variable does not add advantages on the method’s results, and consequently, a simple external calibration approach may be successfully used instead of an IS addition method for quantification purposes. It is also worthwhile to note that the back-interpolation of the experimental values in the regression equations leads to percent biases (Bias %) for the resulting concentration values included in the accepted intervals (+/−15% at all concentration levels except LLOQ where +/−20% is deemed acceptable).

The first attempts of extracting analytes from the urine matrix in both neat *n*-octanol or SUPRAS alternatives led to extremely low extraction yields together with a high level of the residual extracted matrix. Thus, in the case of urine samples, it seemed that controlling the pH of the extracted phase became mandatory. Spiked urine samples were treated prior to extraction with 10 mM heptane-1-sulfonic acid sodium salt (HSNa) and 0,1% formic acid and alternatively with 126 and 252 mM Na_2_CO_3._ Ion pair formation was seen to increase the level of the residual co-extracted matrix, thus increasing interferences. The basic pH produced in the urine phase through the addition of Na_2_CO_3_ leads to a significant increase in the extraction yield and reduction of the co-extracted residual matrix. Doubling the concentration of Na_2_CO_3_ in the urine sample does not add any advantage in terms of extraction yields or reduced levels of the co-extracted residual matrix.

#### 2.3.2. In-Situ SUPRAS Formation

The second sample preparation alternative is the in-situ formation of supramolecular structured phases (SUPRAS) [34,35]. The coacervate separated from water (provided by the biological matrix)/*n*-octanol/water-miscible organic solvent (selected between tetrahydrofuran—THF, ethyl lactate—EL, and *i*-propanol *i*-PrOH) produced good extraction yields of analytes situated in a wide polarity range, with polar analytes being extracted in the aqueous phase formed within the reversed micelles, and apolar analytes being extracted by the hydrophobic walls of the reversed micelles [36]. The volume of the separated SUPRAS phase is smaller compared to the initial volume of the aqueous phase, thus producing a concentrating effect. A simple question pertains to the LC system’s tolerance to injecting increased volumes of SUPRAS. As illustrated in Supplementary Materials, Figure S3, SUPRAS injection volume maxima are 50 μL for plasma/*n*-octanol/*i*-PrOH, 20 μL for plasma/*n*-octanol/EL, and 10 μL for plasma/*n*-octanol/THF. On exceeding these limits, significant peak shape deformations are produced due to sample-focusing phenomena. 

### 2.4. Method Selectivity

**Figure 3:**
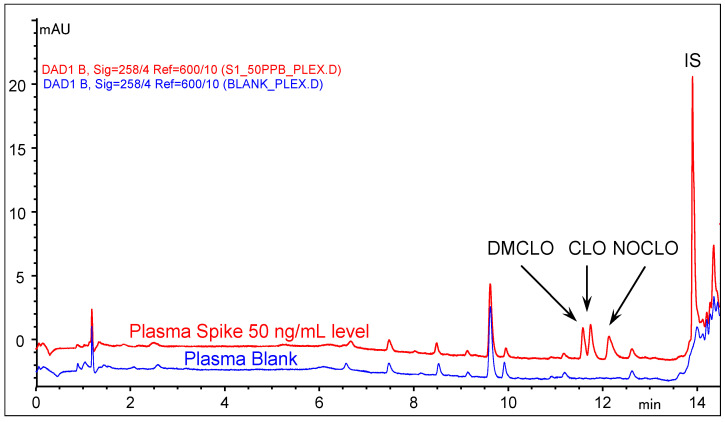
Overlaid chromatograms of a pooled blank plasma sample and a spiked corresponding matrix (50 ng/mL level for DMCLO, CLO, and NOCLO) extracted with *n*-octanol according to conditions described in Section 4.

**Figure 4:**
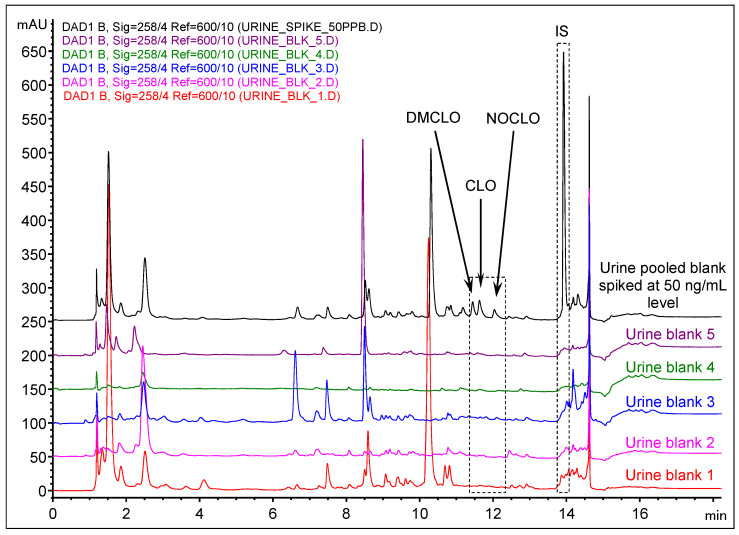
Overlaid chromatograms resulting from LLE extraction of five different urine blank samples (conditions described in Section 4) and a pooled blank sample spiked with 50 ng/mL of target analytes.

Once the sample preparation method and separation conditions were decided, the evaluation of the intrinsic selectivity of the proposed method was mandatory. Processed blank plasma and urine samples were compared with the spikes made at LLOQ for the targeted compounds and IS. The results are illustrated in Figure 3 and Figure 4.

Within the range of elution of the analytes and IS, the co-extracted matrices do not interfere. The plasma blank sample originates from six pooled specimens, collected pre-dose and used previously for bioequivalence studies carried out in LaborMed Pharma bioanalytic laboratory, kept frozen at −80 °C (previous studies received approval from the Romanian National Drug Agency and the Ethics Committee and were carried out according to World Medical Association Declaration of Helsinki). The pooled plasma sample was considered as a worst-case scenario given that it had been collected and used in the past 10 years, and it can be representative of the variability of possible interfering patterns. Five different urine blank samples were freshly collected and stored at −20 °C freezing conditions before use.

### 2.5. Response Functions

Response functions were studied for the target compounds in spiked blank plasma and urine samples, covering the concentration intervals from 50 ng/mL to 1000 ng/mL. Eight concentration levels were considered, and six independent replicates were prepared for each concentration level. Figures S4–S9 (Supplementary Materials) illustrate the response functions produced for all target compounds on extraction from plasma and urine matrices. Both regular and weighed (1/x^2^) linear fit models were applied, using as dependent variables either the analyte peak areas or the peak area ratios between the analyte and IS. The plots presented in the Supplementary Materials Section indicate the normal variation interval of the dependent variable at each concentration level, shown between +/−2 s interval, where s is the standard deviation value calculated from replicates at each concentration level. Repeatability values (RSD%) measured at each concentration level are within the accepted intervals (15%, except for the LLOQ, where 20% is accepted). 

To confirm the determined correlation coefficients, the experimental values were back-interpolated using the regression equations, with percent biases then calculated between back-interpolated and known concentration values. Table 3 lists the determined response function characteristics for the targeted compounds spiked in plasma and urine blank matrices.

Analyzing the data in Table 3, one can observe that (a) LLOQ values are below or close to the 50 ng/mL level; (b) applying weighted linear regression does not improve the LLOQ levels; (c) the use of analyte/IS peak area ratio as the dependent variable instead of the peak analyte area value does not necessarily improve the characteristics of the response function; in the real practice, the external calibration model can successfully replace the IS model; (d) % bias levels outside the +/−15% interval are represented by LLOQ level (+/−20% interval is allowed); the two instances of DMCLO and CLO in plasma matrix (denoted in Table 3 with bold and italic characters) could be solved either by applying the 1/x^2^ weighed linear regression or by the simple elimination of the respective level from the calculation, as long as the situation did not occur at LLOQ or ULOQ levels.

As an additional verification of the repeatability of the extraction process with respect to the IS, in the Supplementary Materials Section, Figure S10, the variation of the IS peak area values recorded during the analysis of the spiked plasma/urine samples produced during the response function study is illustrated. One can observe that the RSD% values are always within the 15% allowed interval and that no trend of variation could be observed (the linear regressions applied to the experimental peak area values plotted in the order of injection are characterized by determination coefficients close to zero, meaning their random distribution against the mean, the slope is close to 0 and the intercept is close to the mean values of the integrated values, all these facts together pleading for the lack of a trend in variation). Linear calibration, with and without the IS, was used as a model for the following experiments (recovery and stability).

### 2.6. Analyte Recovery

Target analyte recovery from plasma and urine matrices in *n*-octanol was studied at four different levels of concentration (50 ng/mL—the LLOQ level; 150 ng/mL—3 × LLOQ; 500 ng/mL—medium level—MQC; 1000 ng/mL—ULOQ level). The study was performed by comparing pre- and post-spiked matrix samples.

For plasma samples, post-spikes were done by extracting 1 mL of a blank sample with 0.5 mL of *n*-octanol. After centrifugation, 295 μL of the organic phase was collected into a glass insert. This aliquot was spiked with 3 μL of analyte working solutions and with 2 μL of the IS working solution, both in *n*-octanol, for a total volume of 300 μL. Handling microliter volumes of solutions was made with 10 μL chromatographic glass syringes. To compensate for the incomplete removal of the organic phase and the concentrating factor produced during LLE extraction, working solutions of the analytes used for post-spiking had individual concentrations of 14.25, 42.75, 142.5, and 285 μg/mL. The IS stock solution was of 750 μg/mL. Three replicates were made for each concentration level. Table S1 (Supplementary Materials) presents the computed recovery values (%) for the target analytes (the peak area value at each concentration level represents the mean value obtained from three replicates). The recoveries of target compounds on extraction from the plasma matrix are around 60%; most likely, the remaining amounts of each compound are entrapped within the interstitial layer. The experimental variability is within acceptance criteria (15% for all concentration levels). Surprisingly, the mean recovery of the IS from the plasma matrix is only 3.2% (RSD of 10%). This could mean that the extraction of the IS in the presented extraction conditions for plasma samples is highly unpredictable, which is, however, contradictory to the results illustrated in Figure S10A (Supplementary Materials). Certainly, the recovery of the IS and target compounds could likely be improved by increasing the pH of the plasma matrix before extraction. However, we considered that the best analytical procedures would have minimal processing of the biological sample before extraction unless certain steps become imperative.

For urine post-spikes, 1.5 mL of blank matrix (with 126 mM Na_2_CO_3_) was extracted with 0.25 mL of *n*-octanol. The experiment was duplicated. From the two obtained organic layers, a total of 295 μL were transferred in a glass insert (500 μL capacity). This aliquot was spiked with 3 μL of analyte working solutions and with 2 μL of the IS working solution, both in *n*-octanol, for a total volume of 300 μL. Handling microliter volumes of solutions was made with 10 μL chromatographic glass syringes. To compensate for the incomplete removal of the organic phase and the concentrating factor produced during LLE extraction, working solutions of the analytes used for post-spiking had individual concentrations of 30, 90, 300, and 600 μg/mL. The IS solution was 300 μg/mL. Table S1 (Supplementary Materials) shows that analyte extraction yields from alkalinized urine matrix are nearly quantitative (92.2% for CLO, 109.9% for CLO, and 94.7% for NOCLO) and the variability is below the maximum accepted level of 15%. The extraction yield for the IS was 69.7% (RSD of 6.3%).

### 2.7. Matrix Effects

We believe that the procedures for evaluating the matrix effects in bioanalytical applications were developed for the use of MS or MS/MS detection in order to evaluate the effects of the co-extracted and co-eluted residual matrix on the ionization yields of the target compound(s). For UV detection, the comparison between the peak areas of the analyte in post-spiked samples and solutions made in neat extraction solvent only illustrates the effect of the interference (co-elution) of the residual matrix on the target analyte. This aspect was already presented in Section 2.4.

However, we decided to apply the matrix effect process to the data produced during method validation. Results are presented in the Table S2 (Supplementary Materials). To compensate for all the corrections made for the post-spiked sample, in the case of comparisons to plasma, solutions in *n*-octanol had concentrations of 142.5, 427.5, 1425, and 2850 ng/mL and 300, 900, 3000, and 6000 ng/mL for urine matrix comparisons. From Table S2, the computed matrix effects are close to 1, meaning that no significant interference (co-elution) arises during the chromatographic separation produced by the co-extracted residual matrix.

### 2.8. Stability

The stability of the stock solutions made in DMSO at different concentration levels when kept in the refrigerator (4 °C) is presented in Supplementary Materials, Table S3. The stability of the stock solutions over a 78-day period at 4 °C was thus proven.

In Tables S3 and S4, the following stability data are also available: (a) long-term stability of the analytes in the biological matrices when kept at −20 °C during a period of 168 h (7 days); this interval was considered sufficient for the declared therapeutic monitoring purposes; (b) stability in the biological matrices at room temperature (25 °C) for 24 h; (c) stability of three consecutive freeze and thaw cycles; (d) stability of the processed samples at room temperature (25 °C) for 48 h. The stability study was performed at four concentration levels (50, 150, 500, 1000 ng/mL) for the target analytes as well as for the IS.

From Tables S3 and S4, one can observe no concerns about the stability of the analytes in the given conditions or over the studied periods, with a single exception: DMCLO in the extracted samples from the urine matrix at room temperature. This finding is difficult to explain, as the stability of the analyte in the unprocessed sample was proven over 24 h. We suspected adsorption on the glass walls, but experiments could not confirm this supposition. In the absence of a logical explanation for this observation, one can state that *n*-octanol extracts from the alkalinized urine matrix should not be kept at room temperature for more than 12 h before analysis due to the significant reduction of the recovered quantity of DMCLO (which is the most probable compound to be detected in such a matrix).

## 3. Discussion

The main scope of this work was to keep experimental conditions as simple as possible in order to make the method affordable for analytical facilities with medium instrumental capabilities.

To keep chromatographic separation conditions straightforward, the additive used in the mobile phase was perchloric acid (0.1% in both aqueous and organic components). The use of perchloric acid, well known for its chaotropic behavior, was preferred for controlling peak symmetry instead of using ion pairing (IP) separation mechanisms frequently chosen in pharmaceutical separation applications. Avoiding IP allows the use of various gradient elution profiles and facilitates different sample preparation approaches without having to carefully match the sample preparation media to the composition of the mobile phase or having important practical limitations of the sample injected volume.

Our approach was to achieve adequate sensitivity by maximizing the sample injection volume and introducing a sample preconcentration stage during the preparation procedure.

The sample preparation alternatives taken into consideration were focused on liquid-liquid extraction (LLE), being robust and affordable procedures which do not require the use of expensive or complicated equipment.

Additionally, we focused on finding an alternative to internal standards belonging to the class of active ingredients used in neuro-psychiatric therapy in order to eliminate the risk of interferences in the real samples resulting from the polypharmacy of potential subjects. 

The analytical decisions for this study were made considering the available literature data about CLO and related metabolites analysis, focusing on HPLC-UV approaches. Table S6, Supplementary Materials, lists the characteristics of the analytical alternatives already existing in the literature. The related references are included in the Supplementary Materials files for the reader, as not all of these references were used in the main body of the manuscript. Based on Table S6, the following conclusions can be made:

(i) only one publication focused on urine as a biological matrix.
(ii) one material used ion-pairing as a separation mechanism, while another used normal phase (NP); all other LC applications used reversed-phase (RP) separations.
(iii) most applications used complex additives in the aqueous component of the mobile phase (inorganic or organic buffers); a few preferred high alkaline pH values (>10), potentially reducing the life span of silica-based stationary phases.
(iv) the sample preparation approaches were tedious, expensive, or both; generally, when LLE was used, the organic phase was usually back-extracted in an acidic aqueous phase (more often 0.1 N HCl), followed by evaporation of the aqueous phase and reconstitution. Sometimes, re-extraction of the aqueous layer with another organic phase, followed by evaporation and reconstitution, was also employed. Automated, online, or manual solid phase extraction (SPE) was often used. In GC applications, an SPME procedure was evaluated. Digitally controlled microextractions by packed sorbent (MEPS), microextraction by packed column procedures, or membrane-assisted solvent extraction (MASE) were also used for sample preparation. Although MS or MS-MS detection offers multiple ways of tuning selectivity and sensitivity, the sample preparation for these methods remained complicated or required costly equipment, whether GC or LC chromatographic approaches were chosen.
(v) in some instances, better quantification limits were obtained; however, most of the studies referred to the determination of pharmacokinetic parameters rather than therapeutic drug monitoring (TDM), which naturally imposed improved sensitivity limits.
(vi) surprisingly, most LC approaches were isocratic; in bioanalytical applications, isocratic elution modes should be avoided due to the potential for accumulating residual matrix components within the column.

To conclude, none of the literature data offered a simple, direct, and straightforward way of sample preparation (simple LLE procedure followed by direct injection of the organic phase). LVI of samples dissolved in solvents non-miscible with the mobile phase or in-situ formation of SUPRAS for extraction and analysis of CLO and related metabolites from either plasma or urine were not previously reported in the literature. The composition of the mobile phase was kept as simple as possible without any compromise in terms of separation selectivity, peak symmetry, or the stability of the stationary phase. 

The proposed analytical method allows the quantitative determination of clozapine and related metabolites (desmethylclozapine and N-oxide clozapine) in the concentration interval ranging from 50 to 1000 ng/mL and is designed for therapeutic monitoring purposes [36].

Diode array detection was used to qualitatively assess the structural confirmation of the target compounds in the spiked biological samples. For illustration purposes, Figure S11 in Supplementary Materials shows the UV spectra of the chromatographic peaks apex of each target analyte from spiked biological matrices (LOQ level) overlaid with the spectrum resulting from the injection of a sample containing the same compounds dissolved in neat *n*-octanol. UV-collected spectra may help structural confirmations of the quantified compounds and sustain the intrinsic selectivity of the analytical approach. 

The sample preparation procedure is based on LLE in *n*-octanol, followed by a large volume injection (50 μL) of the organic phase directly onto the chromatographic column operated under RPLC separation mechanism conditions. According to the author’s experience regarding large-volume injections (LVI) of samples dissolved in solvents that are not miscible with the mobile phase in RPLC [37,38,39,40,41], injection volumes of *n*-octanol as high as 100 μL are feasible. LVI of non-miscible solvents may be assimilated with an online supported liquid extraction (SLE)-RPLC [41]. Accordingly, on increasing the injected volume, an observable effect is the reduction of the retention time, which does not adversely affect the apparent chromatographic resolution, peak shapes, or apparent efficiencies. It is also mandatory to ensure that after each injection, the immiscible solvent plug that forms within the column is eliminated. The gradient elution profile was also adapted to allow the removal of the *n*-octanol solvent plug from the column, leading to an increase of around 10 min of the chromatographic run. However, this organic (acetonitrile) column wash confers additional benefits as this can also prevent the accumulation of apolar residual matrix components co-extracted from the biological matrices in the column. Considering that this analytical solution is designed for therapeutic monitoring, meaning that the number of samples is moderate, the length of the chromatographic run can be considered acceptable.

The effect of injected volume on retention is illustrated in the Supplementary Materials, Figures S12 and S13, with the lowest retention reduction observable for the IS, as it elutes closely before the start of the *n*-octanol front. Once the sample plug is transferred to the column, due to the increased viscosity of *n*-octanol, a pressure pulse is observed, with the pressure drop returning to the initial value only after the expansion of the *n*-octanol plug and subsequent penetration of the mobile phase breakthrough it. Figure S14 in the Supplementary Materials Section illustrates the additional pressure pulse produced by increasing the injection volume. To keep the method applicable to normal HPLC systems (max. operating pressure of 400 bar), a maximum injection volume of 50 μL seems to be the safest approach. As such, an overall concentrating factor of 100 may be obtained from increasing the injection volume (50 folds) and from the LLE (2 folds). Additionally, former experience with *n*-octanol extraction from plasma [36] showed that the separation between the two layers (aqueous and *n*-octanol) is produced through an interstitial mechanically resistant layer, allowing good isolation of the organic upper layer. This, however, also involves some organic solvent consumption in the formation of this layer, which improves the concentrating factor (see Supplementary Materials, Figure S15). 

The peak symmetry of the analytes was controlled by adding 0.1% HClO_4_ in both mobile phase solvents. Perchloric acid acts as a chaotropic additive, achieving a fair elution profile without complicating the mobile phase composition, such as the addition of inorganic or organic buffers to ensure strict pH values or ion pairing agents in the case of IP-RPLC separations.

Keeping the plasma sample pH at physiological levels allows the extraction of analytes with yields placed in the 60% interval. The loss may be compensated by the reduction of the volume of the extractant phase by a factor of 2 with respect to the volume of the biological sample. In the case of urine matrices, pH control before extraction is mandatory. Creating an alkaline pH by means of Na_2_CO_3_ addition drastically increases the extraction yields and reduces the pattern of the residual co-extracted matrix. We used Na_2_CO_3_ instead of NaOH, as existing literature [26,27,29,42] indicated that sodium hydroxide can lead to the retro-conversion of the N-oxide metabolite to clozapine. Making the aqueous phase alkaline prior to processing the samples allowed quantitative extraction yields and significantly reduced the residual co-extracted matrix.

An alternative sample preparation procedure being tested referred to the in-situ formation of SUPRAS from ternary systems, with water provided by the biological sample/*n*-octanol-acting as surfactant/water-miscible organic solvent chosen between tetrahydrofuran, ethyl lactate or *iso*-propyl alcohol-to produce coacervation. Good extraction yields were obtained, especially when extracting alkalinized urine samples. However, the major drawback of the SUPRAS alternative was the limitation on the maximum injection volume (to 20 or even 10 μL) to avoid peak shape distortions and the relatively high amount of residual co-extracted matrix, altering the method’s selectivity. Considering that increasing the injection volume is one important way to increase a method’s sensitivity, the alternative of using in-situ formed SUPRAS to isolate the target analytes was found unsuitable for the scope of this analytical approach. However, given the somewhat customizable nature of SUPRAS, further research should also focus on developing a mixture that would effectively extract and concentrate these target analytes from plasma and urine without over-extracting residual matrix components.

Both spiked matrix calibrations indicate that using only analyte peak areas can potentially produce better results than the IS-based methodology. Nonetheless, given the possible errors introduced via sample processing due to instruments and tools (e.g., microliter pipette volume variations) or between personnel, the more conservative peak ratio method should still be preferred. 

The performed stability studies (long-term stability in the matrix at −20 °C for 7 days, short-term stability in the matrix at 25 °C for 24 h, stability against three successive freeze and thaw cycles, and processed sample stability at room temperature for 48 h) indicate that analytes were stable in the given conditions. No tendency for back-conversion of the N-oxide metabolite to clozapine was registered. Surprisingly, the desmethyl metabolite extracted from urine had a reduced stability in the processed sample at room temperature. To ensure the stability of DMCLO, the period between the end of the extraction process from the urine matrix and the start of the analysis should not be higher than 12 h.

When applying the method to real plasma and urine patient samples, evaluation of the interfering potential of other drugs traditionally administered alongside clozapine or medication commonly prescribed for other illnesses (e.g., diabetes, thyroid affections, etc.) may be necessary.

## 4. Materials and Methods

### 4.1. Materials

The following reagents and chemicals were used for this project: clozapine and metabolites (clozapine N-oxide and desmethylclozapine) (Sigma-Aldrich, Saint Louis, MO, USA), heptane-1-sulfonic acid sodium salt (Merck LiChropur^®^, Merck KGaA, Darmstadt, Germany), sodium carbonate (Merck), aniline yellow (Gurr), *n*-octanol (Emplura^®^, ≥99%), acetonitrile (Sigma-Aldrich Chromasolv^®^ gradient grade, for HPLC, ≥99.9%), tetrahydrofuran (Merck, pro analysis grade, ≥99.5%), formic acid (ULC/MS grade 99%, Biosolve Ltd., Dieuze, France), perchloric acid (Sigma-Aldrich, puriss. p.a.), dimethylsulfoxide (Fluka Chemicals, Geel, Belgium), isopropanol (2-propanol, VWR International, AnalaR NORMAPUR, West Chester, PA, USA), (−)-ethyl L-lactate (Fluka, photoresist grade, ≥99.0%). HPLC grade water (resistivity ≥ 18.2 MΩ × cm, TOC ≤ 10 ppb, bacterial count ≤ 10 CFU/mL) was produced by a Milli-Q Integral S system.

Plasma blank samples originate from pooled specimens (collected pre-dose) representing counter samples produced during bioequivalence studies carried out in the past 10 years in LaborMed Pharma bioanalytic laboratory kept in freezing conditions at −80 °C (all these previous studies received approval from the Romanian National Drug Agency and the Ethics Committee and were carried out according to World Medical Association Declaration of Helsinki). After spiking, plasma samples were kept in freezing conditions at −20 °C until the processing stage.

Urine samples were collected from the author’s team members in sterile plastic containers and kept before any spiking and/or processing stages in freezing conditions at −20 °C. Pooled urine specimens from the individual collected samples were used during the method’s validation, except for the Selectivity stage.

### 4.2. Equipment and Chromatographic Column

An Agilent 1260 series system (Agilent Technology, Santa Clara, CA, USA) with a Zorbax Eclipse XDB–C18, 4.6 × 150 mm, 5 microns (Agilent) column was used for the chromatographic separations. The system is equipped with a quaternary pump with a four channels degasser (G1311B), an automated liquid sampler fitted with a 100 µL injection loop (G1329B), a column thermostat (G1316C), and a multi-channel diode-array detector (G1365D). Agilent ChemStation software LC3D, version 04.03(16), was used for hardware control data acquisition and processing.

An Eppendorf MiniSpin 5452 centrifuge from Eppendorf AG (Hamburg, Germany) was used for phase separation in LLE extraction processes. A Stuart vortex mixer SA8 model (Sigma-Aldrich) was used for sample homogenization, as well as a 2200 ETH S3 Sonica ultrasonic cleaner (Soltec). 

### 4.3. Chromatographic Parameters

The chromatographic separation used a mobile phase consisting of component A representing an aqueous 0.1% (*v*/*v*) HClO_4_ solution and component B representing ACN with the addition of 0.1% (*v*/*v*) HClO_4_. A flow rate of 1.5 mL/min, column temperature of 25 °C, and 50 µL injection volumes were used. Table 4 represents the gradient profile used for the chromatographic separation, including the column re-equilibration stage. The acquisition wavelength was 258 ± 4 nm, with a reference wavelength of 600 ± 10 nm, an acquisition frequency of 5 Hz, and a resolution of 2 nm.

### 4.4. Computation of LLOQ Values

LLOQ values were computed according to the relationship $LLOQ=\frac{t\times \left(s_{A}+\bar{x}\times s_{B}\right)}{B+2\times t\times s_{B}}$, where B is the slope of the linear regression, *s_B_* is the standard deviation of the slope, *s_A_* is the standard deviation of the intercept, $\bar{x}$ is the mean concentration used during calibration (independent variable), and t is the two-sided Student distribution coefficient, considered for a probability P% = 99% and (n-2) degrees of freedom, n being the number of the calibration levels [43].

### 4.5. Stock and Working Solutions

Individual stock solutions of clozapine (2200 ppm), desmethylclozapine (1000 ppm), and clozapine N-oxide (1000 ppm) were prepared in DMSO. From these, eight intermediate calibration concentrations containing the three analytes were prepared in DMSO at 5, 10, 12.5, 15, 20, 25, 50, and 100 ppm. A stock DMSO solution of aniline yellow (10,000 ppm) was prepared to be used as an internal standard.

For calibration purposes, sample spikes of both urine and plasma were prepared by a 1:100 dilution of the intermediate concentrations in a biological matrix to a volume of 10 mL, resulting in a 50–1000 ppb calibration range. The IS added to the samples was matrix-dependent, with 10 µL added to plasma samples and 2 µL to urine samples.

### 4.6. Sample Preparation Stages

LLE procedure of plasma samples was achieved through transferring 1 mL aliquot to an Eppendorf tube of 2 mL capacity and addition of 0.5 mL of *n*-octanol. Following a vortex mixing period of 3 min at 2500 rpm, the samples are centrifuged at room temperature with a relative centrifugal force (RCF) of 7560× *g* for 20 min. The supernatant phase (300 μL) was collected by means of a 100 μL volume glass chromatographic syringe (Roth) and transferred in an HPLC glass vial fitted with a 500 μL glass insert. Note that the 300 μL of the *n*-octanol phase does not quantitatively remove the extractant. It was determined that the whole volume of the *n*-octanol phase separated in the process is closer to 0.35 mL. Collecting the supernatant only partially eliminates the possibility of incidentally collecting some of the aqueous phases. 

Prior to LLE extraction, during the spiking step, 134 mg of Na_2_CO_3_ (corresponding to a concentration of 126 mM) are weighed and added to the 10 mL volumetric flasks used for spiking the urine samples. The optimized LLE procedure used for urine samples is as follows: (a) In an Eppendorf tube of 2 mL capacity transfer a 1.5 mL aliquot of urine sample; (b) add 0.25 mL of *n*-octanol and vortex-mix for 3 min at 2500 rpm; (c) transfer the tubes to the centrifuge, and process at room temperature with a relative centrifugal force (RCF) of 7560× *g* for 20 min; (d) the supernatant phase (200 μL) was collected by means of a 100 μL volume glass chromatographic syringe (Roth) and transferred in a HPLC glass vial fitted with a 500 μL glass insert. 

Evaluation of in-situ SUPRAS formation as an alternative to LLE in *n*-octanol was achieved by means of the following experiments: 0.8 mL spiked blank plasma samples were mixed in a volumetric ratio 1/1 with a SUPRAS forming mix produced between a water-miscible organic solvent and *n*-octanol (4/1 *v/v*, where the water-miscible organic solvent was THF, EL or *i*-PrOH). After a vortex mixing period of 3 min at 2500 rpm, the Eppendorf tube was transferred in the centrifuge operated at 7560× *g* for 20 min. The upper SUPRAS phase was transferred by means of a chromatographic glass syringe in an injection vial. As discussed in Section 2.3, due to limitations to the maximum injected volume of the SUPRAS phase in the chromatographic column, this sample preparation alternative was abandoned.

## 5. Conclusions

An HPLC-DAD method was developed and validated in order to quantitatively determine clozapine and related metabolites (desmethyl and N-oxide) in human plasma and urine matrices. The method achieved therapeutic drug monitoring (TDM) levels between 50 to 1000 ng/mL intervals for all target compounds, with LLOQ values around 50 ng/mL, precision (RSD%) within the 15% range, and accuracies within +/−15%. Spectral confirmation is possible.

Sample preparation was based on LLE in *n*-octanol followed by LVI (50 μL) of the organic layer directly to the chromatographic column. The urine matrix needed alkalinization (through Na_2_CO_3_ addition) before LLE. Extraction of the target analytes in a supramolecular phase (SUPRAS) in-situ formed through using the water from the matrix, *n*-octanol and a polar solvent miscible with water (chosen amidst *i*-propanol, ethyl lactate, and tetrahydrofuran) was also explored. The relatively poor selectivity of such an extraction process and limitations related to the maximum injected volume (max. 20 μL) led us to the decision to abandon this alternative approach, although its intrinsic potential still needs further studies. 

The control of peak symmetry during LC elution was achieved through the simple addition of 0.1% HClO_4_ in both components of the mobile phase, acting as a chaotropic agent. The gradient profile was constructed in order to achieve apparent retention factors higher than 5 for all targeted compounds and to produce the elimination of the *n*-octanol plug after each injection. This feature also limits the eventuality of the hydrophobic residual matrix to accumulate in the column’s head.

While not uncommon, the study’s quantitative aspect demonstrated that the use of an internal standard, in this case, aniline yellow, did not significantly improve the resulting LLOQs. Our choice of the internal standard deviated from the common practice where another drug within the same therapeutic class as the analytes would be used. This was due to clozapine’s potential to be prescribed with other antipsychotic medications, and choosing one such as the internal standard may lead to erratic results when analyzing incurred patient samples.

The presented analytical solution (sample preparation and chromatographic separation taken together) is a relatively cheap and simple alternative for monitoring therapeutic levels of clozapine and related metabolites in complicated biological matrices such as plasma and urine. The quality attributes of the methods were demonstrated through validation, together with the stability of the targeted compounds in the different stages of the sample preparation and different time periods.

## Figures and Tables

**Table 1:** Response function characteristics for DMCLO, CLO, and NOCLO neat solutions in DMSO, considering eight concentration levels ranging from 5 to 100 μg/mL (1 μL injected volume).

Analyte	DMCLO		CLO		NOCLO	
Fit Model	Linear	Weighted 1/x^2^	Linear	Weighted 1/x^2^	Linear	Weighted 1/x^2^
Slope	2.63361	2.58518	2.80215	2.64079	2.38423	2.34487
Intercept	−0.56785	0.17154	−4.19390	−1.03232	−0.43422	0.15129
Correlation Coefficient	0.99979	0.99684	0.99958	0.99735	0.99980	0.99711
LLOQ (ng in column)	1.6	3.4	2.3	3.4	1.6	3.3
Min. % Bias on back interpolation	−12.2	−12.9	−6.9	−10.8	−11.9	−12.4
Max. % Bias on back interpolation	7.0	3.3	20.3	5.8	6.1	2.9

**Table 2:** Response function characteristics for DMCLO, CLO, and NOCLO neat solutions in *n*-octanol, considering eight concentration levels ranging from 50 to 1000 ng/mL (50 μL injected volume).

Analyte	DMCLO		CLO		NOCLO	
Dependent Variable	Peak Area	IS Area Ratio	Peak Area	IS Area Ratio	Peak Area	IS Area Ratio
Linear model						
Slope	0.12368	0.00051	0.14072	0.00058	0.11108	0.00046
Intercept	0.34516	−0.00412	1.79759	0.00055	−1.28965	−0.00973
Correlation coeff.	0.99990	0.99942	0.99882	0.99927	0.99862	0.99783
LLOQ (ng/mL)	12.7	28.7	39.6	31.9	42.5	51.8
min. % Bias	−3.2	−7.8	−20.4	−10.1	−10.4	−12.6
max. % Bias	7.6	20.0	16.6	19.2	3.8	6.0
Weighted model 1/x^2^						
Slope	0.12094	0.00048	0.14838	0.00059	0.09439	0.00038
Intercept	0.84198	0.00157	0.54773	0.00026	1.78105	0.00531
Correlation coeff.	0.99929	0.99680	0.99106	0.99156	0.97909	0.97227
LLOQ (ng/mL)	21.1	36.3	48.7	48.0	59.3	60.8
min. % Bias	−3.0	−7.7	−8.7	−10.5	−23.5	−22.2
max. % Bias	2.9	8.1	19.0	18.8	14.8	19.6

**Table 3:** Response function characteristics for DMCLO, CLO, and NOCLO spiked plasma and urine samples for eight concentration levels ranging from 50 to 1000 ng/mL and six independent replicates per concentration level.

Matrix	Plasma					
Analyte	DMCLO		CLO		NOCLO	
Functional fit	Linear					
Dependent variable	Peak area	Ratio *	Peak area	Ratio	Peak area	Ratio
Slope	0.24524	0.00375	0.27213	0.00416	0.22138	0.00339
Intercept	−1.09179	−0.04445	−0.58657	−0.03985	3.94425	0.03034
Correlation coefficient	0.99958	0.99947	0.99979	0.99874	0.99945	0.99907
LLOQ (ng/mL)	24.8	27.6	17.9	40.9	28.2	35.7
Min. % bias	−3.3	−9.0	−4.7	−9.6	−7.5	−10.9
Max. % bias	9.9	***21.9***	19.3	***31.0***	11.0	18.8
Functional fit	Weighted 1/x^2^					
Slope	0.23792	0.00351	0.25645	0.00378	0.21886	0.00321
Intercept	0.04006	0.00227	2.46537	0.03822	4.23978	0.06502
Correlation coefficient	0.99849	0.99703	0.99872	0.99630	0.99592	0.99526
LLOQ (ng/mL)	28.1	35.4	26.5	38.0	39.1	40.9
Min. % bias	−3.6	−8.0	−4.8	−8.8	−7.3	−10.4
Max. % bias	6.4	5.9	5.4	9.5	11.6	9.3
Matrix	Urine					
Functional fit	Linear					
Slope	0.65408	0.00131	0.79349	0.00161	0.57830	0.00114
Intercept	−5.76703	−0.04266	28.15546	0.00052	−15.1187	−0.04058
Correlation coefficient	0.99928	0.99854	0.99943	0.99874	0.99917	0.99946
LLOQ (ng/mL)	31.80	43.7	28.6	40.8	33.9	27.8
Min. % bias	−10.69	−14.1	−10.0	−9.6	−9.0	−4.9
Max. % bias	8.40	13.3	16.0	19.4	8.3	12.9
Functional fit	Weighted 1/x^2^					
Slope	0.63728	0.00125	0.77798	0.00153	0.56828	0.00110
Intercept	−2.21393	−0.03003	31.53629	0.01528	−13.1219	−0.03329
Correlation coefficient	0.99681	0.99420	0.99402	0.98977	0.99652	0.99774
LLOQ (ng/mL)	36.2	43.4	43.7	50.4	37.2	32.4
Min. % bias	−10.6	−13.9	−11.1	−11.2	−8.8	−5.4
Max. % bias	7.5	6.8	13.9	16.2	7.4	7.2

* Analyte to IS peak area ratio.

**Table 4:** Gradient conditions were used for chromatographic separations.

Time (min)	Mobile Phase A (%)	Mobile Phase B (%)
0	97.5	2.5
4	97.5	2.5
10	70	30
12	70	30
14	0	100
24	0	100
24.01	97.5	2.5
27	97.5	2.5

## Data Availability

The original contributions presented in the study are included in the article/Supplementary Materials, further inquiries can be directed to the corresponding author.

## Supplementary Materials

The following supporting information can be downloaded at: https://www.mdpi.com/article/10.3390/molecules29215039/s1, Figure S1. Calibrations for DMCLO, CLO and NOCLO in DMSO (concentration interval 5–100 μg/mL, 1 μL injected volume). Figure S2. Calibrations for DMCLO, CLO and NOCLO in neat *n*-octanol, 50 μL injection volume. Both linear and weighted 1/x^2^ models were used for detector response fit, with and without consideration of the IS. Figure S3. Overlaid chromatograms of increasing volumes of the extracts from plasma matrices in SUPRAS formed in-situ, based on *iso*-propyl alcohol/ethyl lactate/tetrahydrofuran mixed with *n*-octanol. Figure S4. Calibrations for DMCLO in spiked plasma samples prepared through LLE in *n*-octanol, using either peak area (mAU*s) or analyte/IS peak area ratio (no units). Blue lines illustrate the linear regression model, while red lines represent the weighted 1/x^2^ linear regression model. Vertical bars indicate the normal variation intervals of the mean values obtained from six independent samples analyzed at each concentration level, expressed as +/−2 x standard deviation. Figure S5. Calibrations for CLO in spiked plasma samples prepared through LLE in *n*-octanol, using either peak area (mAU*s) or analyte/IS peak area ratio (no units). Blue lines illustrate the linear regression model, while red lines represent the weighted 1/x^2^ linear regression model. Vertical bars indicate the normal variation intervals of the mean values obtained from six independent samples analyzed at each concentration level, expressed as +/−2 x standard deviation. Figure S6. Calibrations for NOCLO in spiked plasma samples prepared through LLE in *n*-octanol, using either peak area (mAU*s) or analyte/IS peak area ratio (no units). Blue lines illustrate the linear regression model, while red lines represent the weighted 1/x^2^ linear regression model. Vertical bars indicate the normal variation intervals of the mean values obtained from six independent samples analyzed at each concentration level, expressed as +/−2 x standard deviation. Figure S7. Calibrations for DMCLO in spiked urine samples prepared through LLE in *n*-octanol after alkalinization, using either peak area (mAU*s) or analyte/IS peak area ratio (no units). Blue lines illustrate the linear regression model, while red lines represent the weighted 1/x^2^ linear regression model. Vertical bars indicate the normal variation intervals of the mean values obtained from six independent samples analyzed at each concentration level, expressed as +/−2 x standard deviation. Figure S8. Calibrations for CLO in spiked urine samples prepared through LLE in *n*-octanol after alkalinization, using either peak area (mAU*s) or analyte/IS peak area ratio (no units). Blue lines illustrate the linear regression model, while red lines represent the weighted 1/x^2^ linear regression model. Vertical bars indicate the normal variation intervals of the mean values obtained from six independent samples analyzed at each concentration level, expressed as +/−2 x standard deviation. Figure S9. Calibrations for NOCLO in spiked urine samples prepared through LLE in *n*-octanol after alkalinization, using either peak area (mAU*s) or analyte/IS peak area ratio (no units). Blue lines illustrate the linear regression model, while red lines represent the weighted 1/x^2^ linear regression model. Vertical bars indicate the normal variation intervals of the mean values obtained from six independent samples analyzed at each concentration level, expressed as +/−2 x standard deviation. Figure S10. Variation of the IS peak areas values obtained during the response function study on analyzing plasma (A) and urine (B) matrices. Table S1. Target analyte recovery on LLE extraction from human plasma and urine matrices in *n*-octanol. Table S2. Matrix effects on target analytes from LLE extraction of human plasma and urine matrices in *n*-octanol. Table S3. Long-term stability of stock solutions of DMCLO, CLO and NOCLO in DMSO (4 °C). Table S4. Stability data for target analytes and IS in the plasma matrix (recorded peak areas). Table S5. Stability data for target analytes and IS in the urine matrix (recorded peak areas). Table S6. Exhaustive literature data search about clozapine and related metabolites quantitative determination in biological matrices by means of chromatographic methods (focus was made on HPLC-DAD methods). Figure S11. Overlaid UV spectra of the target analytes at the peak apex at LOQ level spiked biological matrices (urine—blue trace; plasma—red trace) compared to the spectra of the analytes in the chromatogram of a neat *n*-octanol solution (green trace). Figure S12. Overlaid chromatograms of increased injection volumes (10–100 μL) of the analytes in *n*-octanol. Figure S13. Apparent retention factor decrease for the analytes with *n*-octanol injection volume increase. Figure S14. Variation of column pressure drop with increased injection volumes of solutions/extracts in *n*-octanol, registered during the sample plug transfer into the column. Figure S15. Layer separation after centrifugation of plasma samples extracted with *n*-octanol. Attention should be paid to the intermediate layer separating plasma from *n*-octanol extract, which makes collection easier, but incorporates some of the extracting solvent. The total *n*-octanol volume recovered after extraction can be fairy approximated to 0.35 mL from 0.5 mL used in the process (around 70%). References [8,11,12,15,16,18,19,22,44,45,46,47,48,49,50,51,52,53,54,55,56,57,58] are cited in the Supplementary Materials.
