# Supplementary material for: A Simple HPLC-DAD Method for the Therapeutic Monitoring of Clozapine and Related Metabolites in Human Plasma and Urine Samples
Source: Molecules. 2024 Oct 25;29(21):5039. doi: 10.3390/molecules29215039 (PMC11547683; doi:10.3390/molecules29215039)
Supplement: Supplementary file 1 [file molecules-29-05039-s001.zip › molecules-3261628-supplementary.pdf]

molecules

Supplementary Materials

A simple HPLC-DAD method for the therapeutic monitoring of clozapine and related metabolites in human plasma and urine samples

Mircea-Alexandru Comănescu, Dana-Maria Preda, Flavian-Ştefan Rădulescu, Victor Voicu and Andrei-Valentin Medvedovici

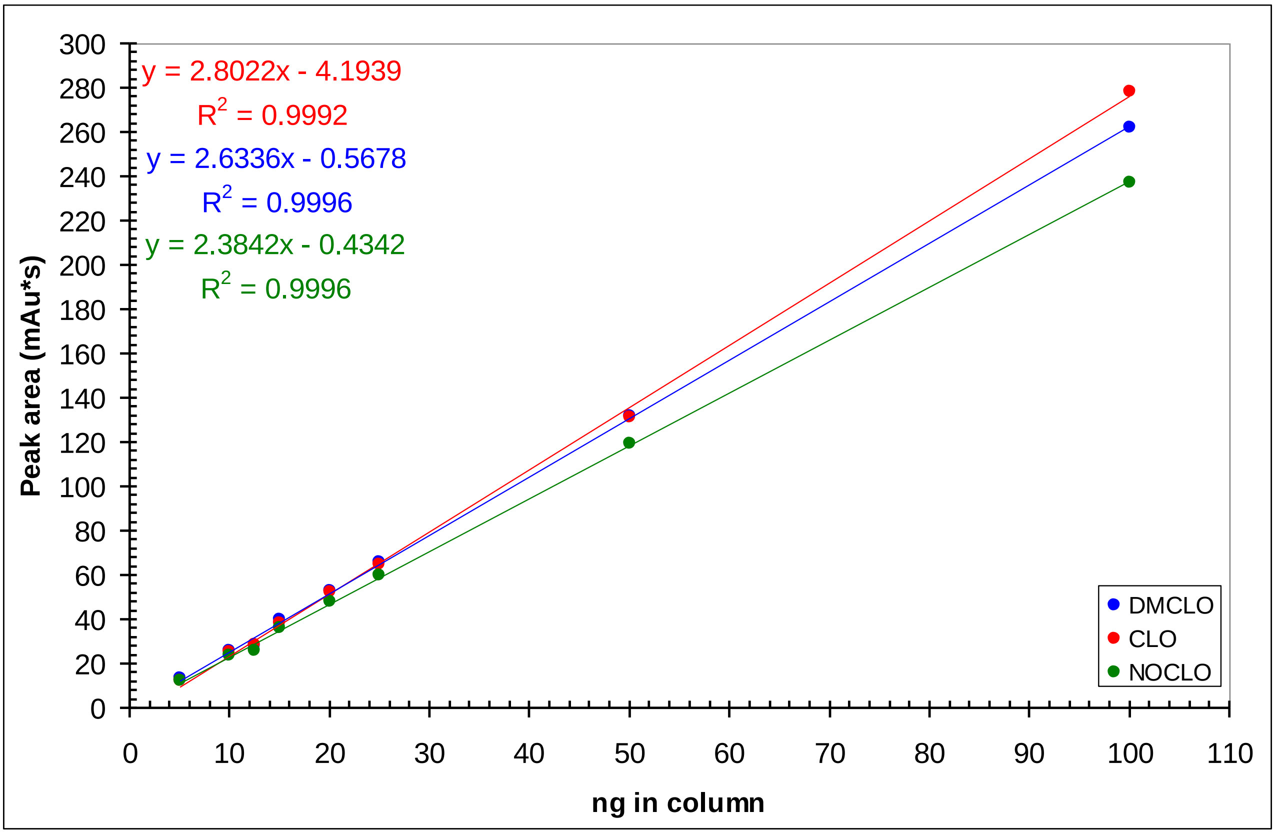

Figure S1 . Calibrations for DMCLO, CLO and NOCLO in DMSO (concentration interval 5 -100 µg/mL, 1 µL injected volume)

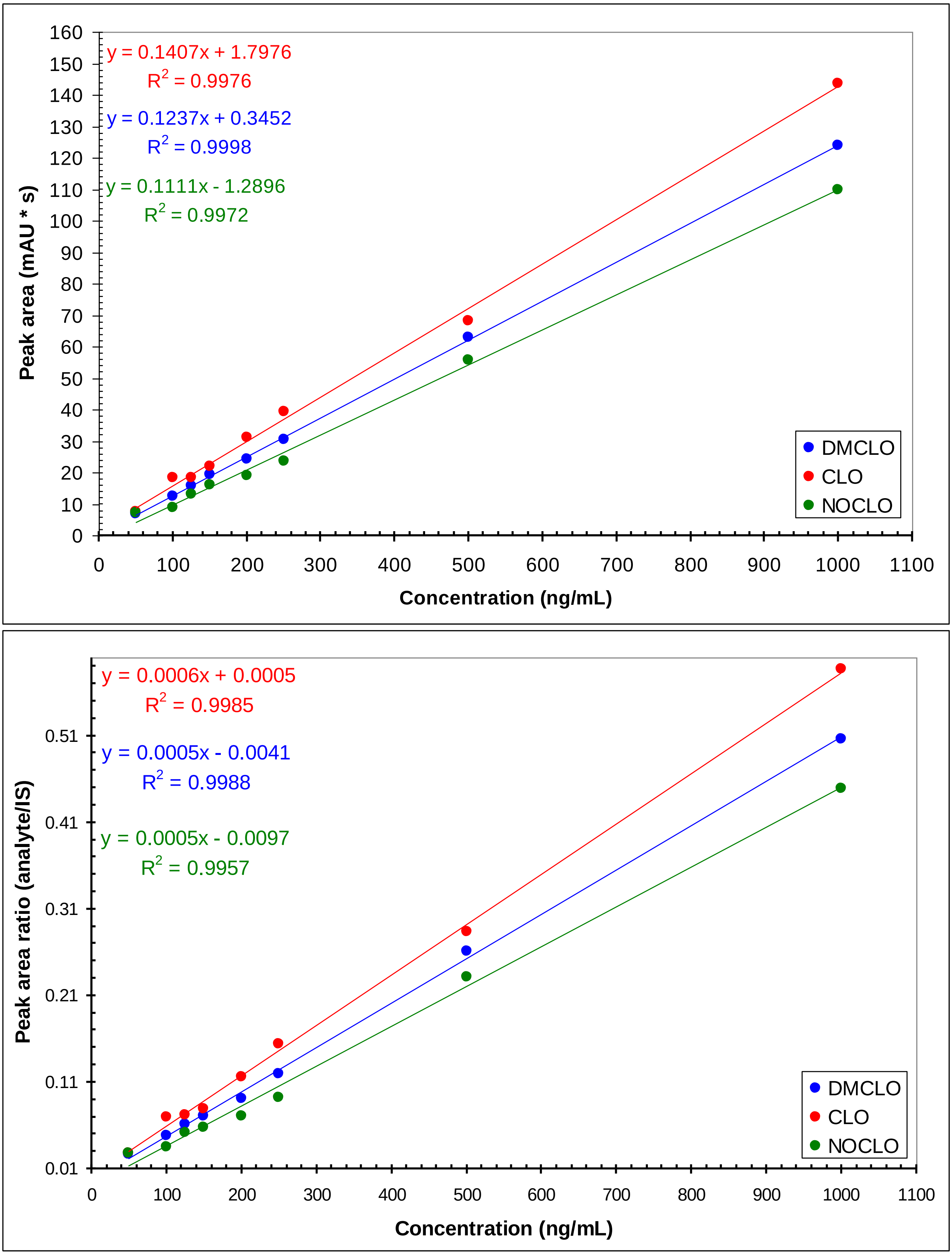

Figure S2. Calibrations for DMCLO, CLO and NOCLO in neat *n*-octanol, 50 µL injection volume. Both linear and weighted $1/x^2$ models were used for detector response fit, with and without consideration of the IS.

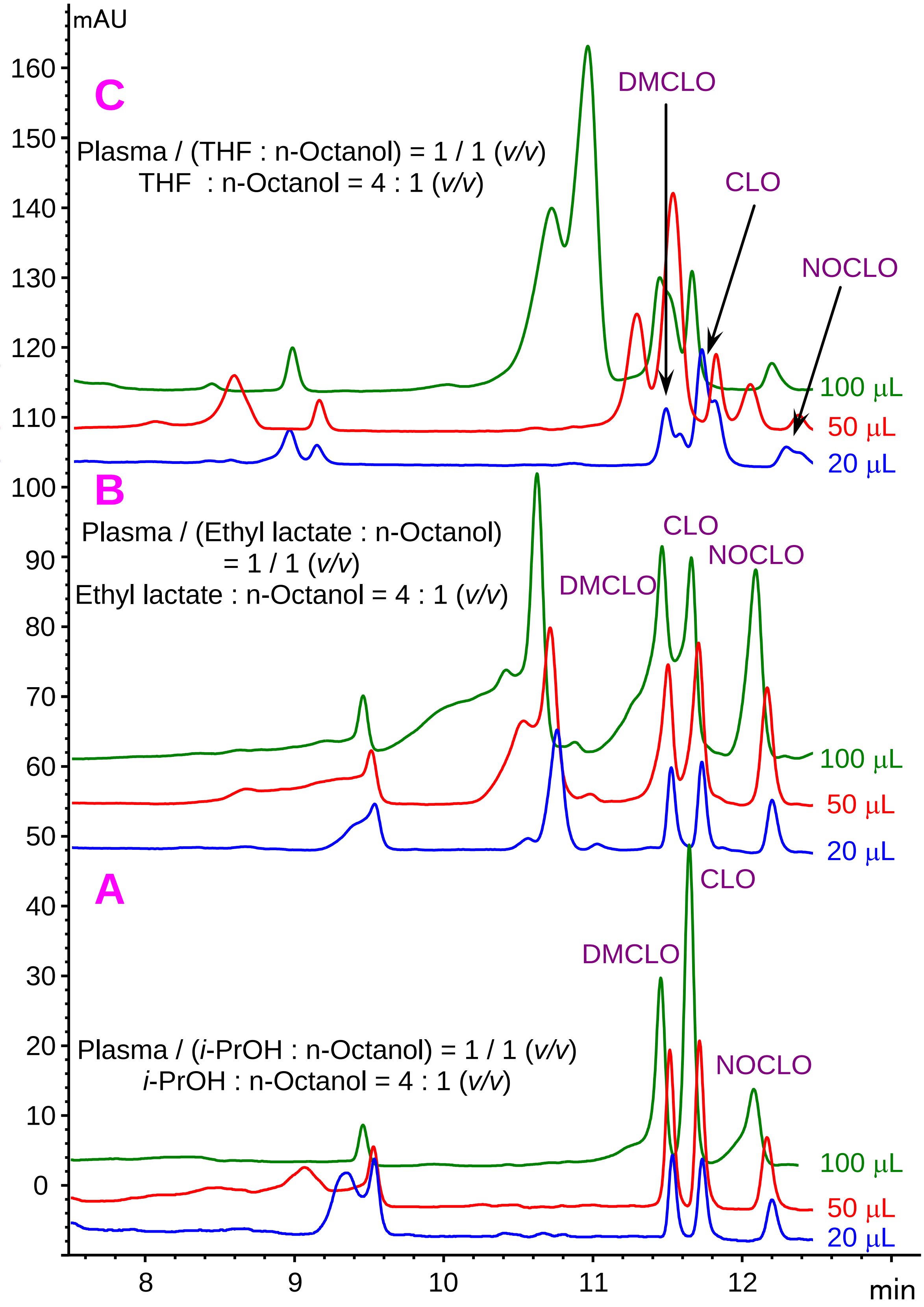

23

Figure S3. Overlaid chromatograms of increasing volumes of the extracts from plasma matrices in SUPRAS formed *in-situ*, based on *iso*-propyl alcohol / ethyl lactate / tetrahydrofuran mixed with *n*-octanol. 24 25

26

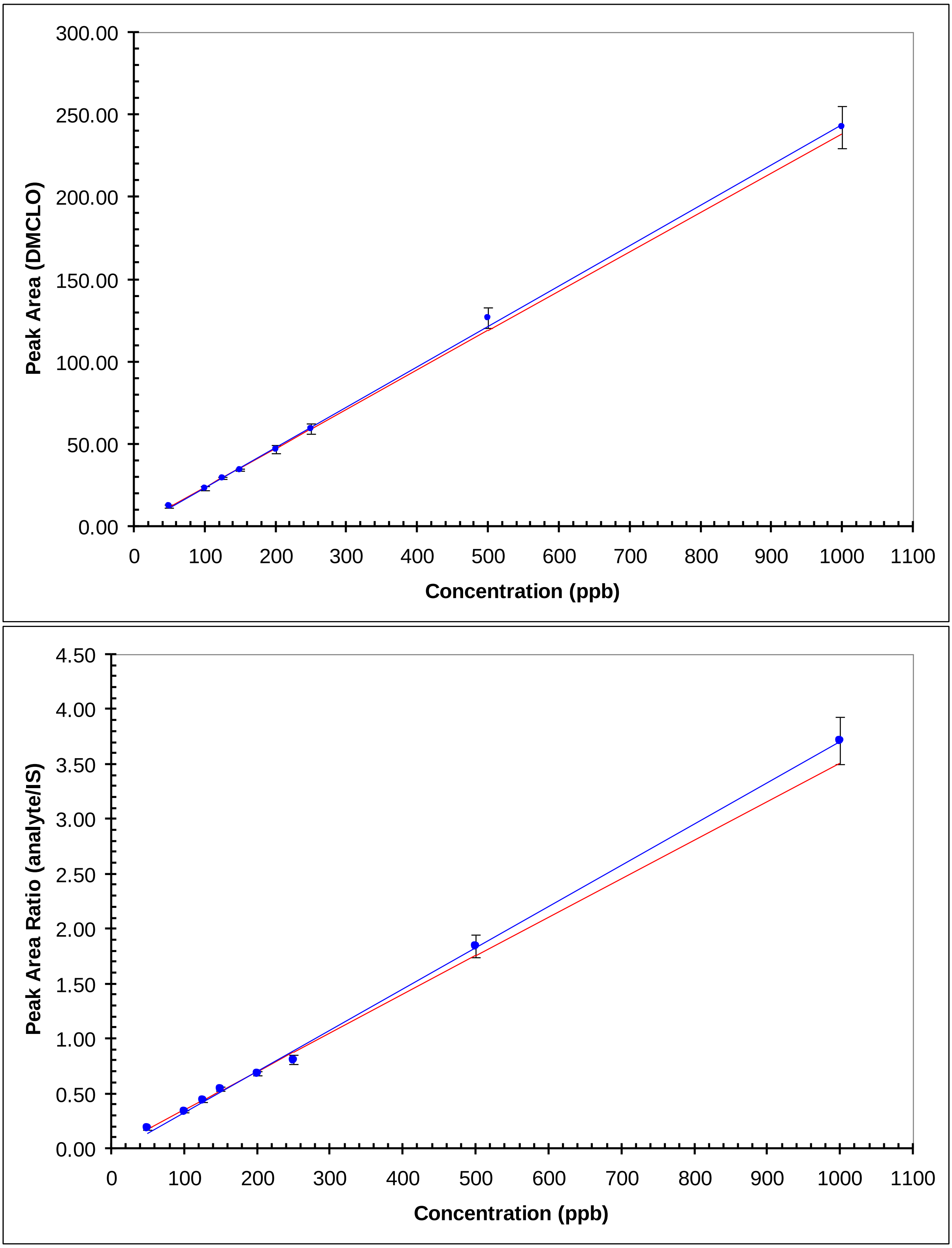

Figure S4. Calibrations for DMCLO in spiked plasma samples prepared through LLE in *n*-octanol, using either peak area (mAU*s) or analyte/IS peak area ratio (no units). Blue lines illustrate the linear regression model, while red lines represent the weighted 1/x^2 linear regression model. Vertical bars indicate the normal variation intervals of the mean values obtained from six independent samples analyzed at each concentration level, expressed as +/- 2 x standard deviation.

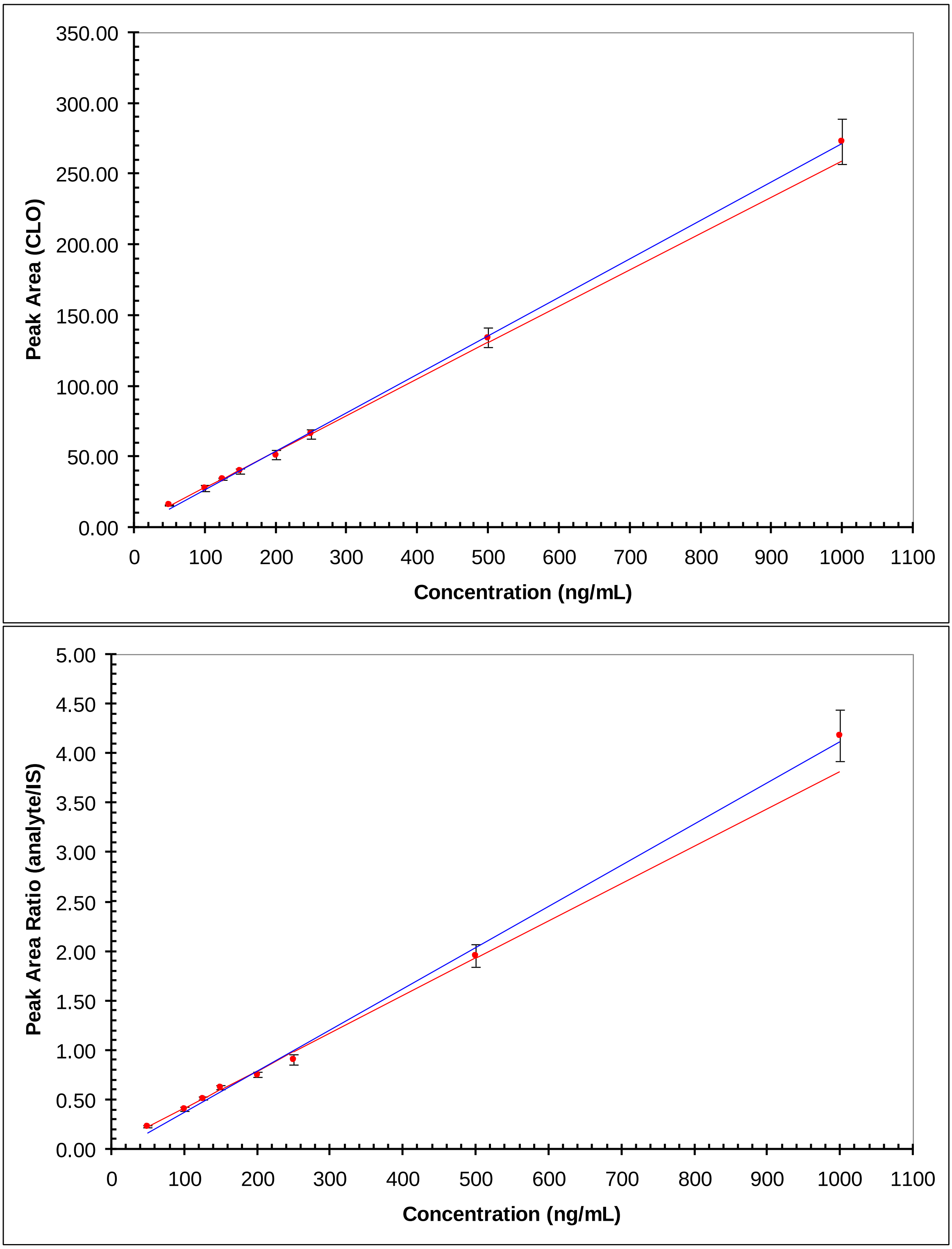

Figure S5. Calibrations for CLO in spiked plasma samples prepared through LLE in *n*-octanol, using either peak area (mAU*s) or analyte/IS peak area ratio (no units). Blue lines illustrate the linear regression model, while red lines represent the weighted $1/x^2$ linear regression model. Vertical bars indicate the normal variation intervals of the mean values obtained from six independent samples analyzed at each concentration level, expressed as +/- 2 x standard deviation.

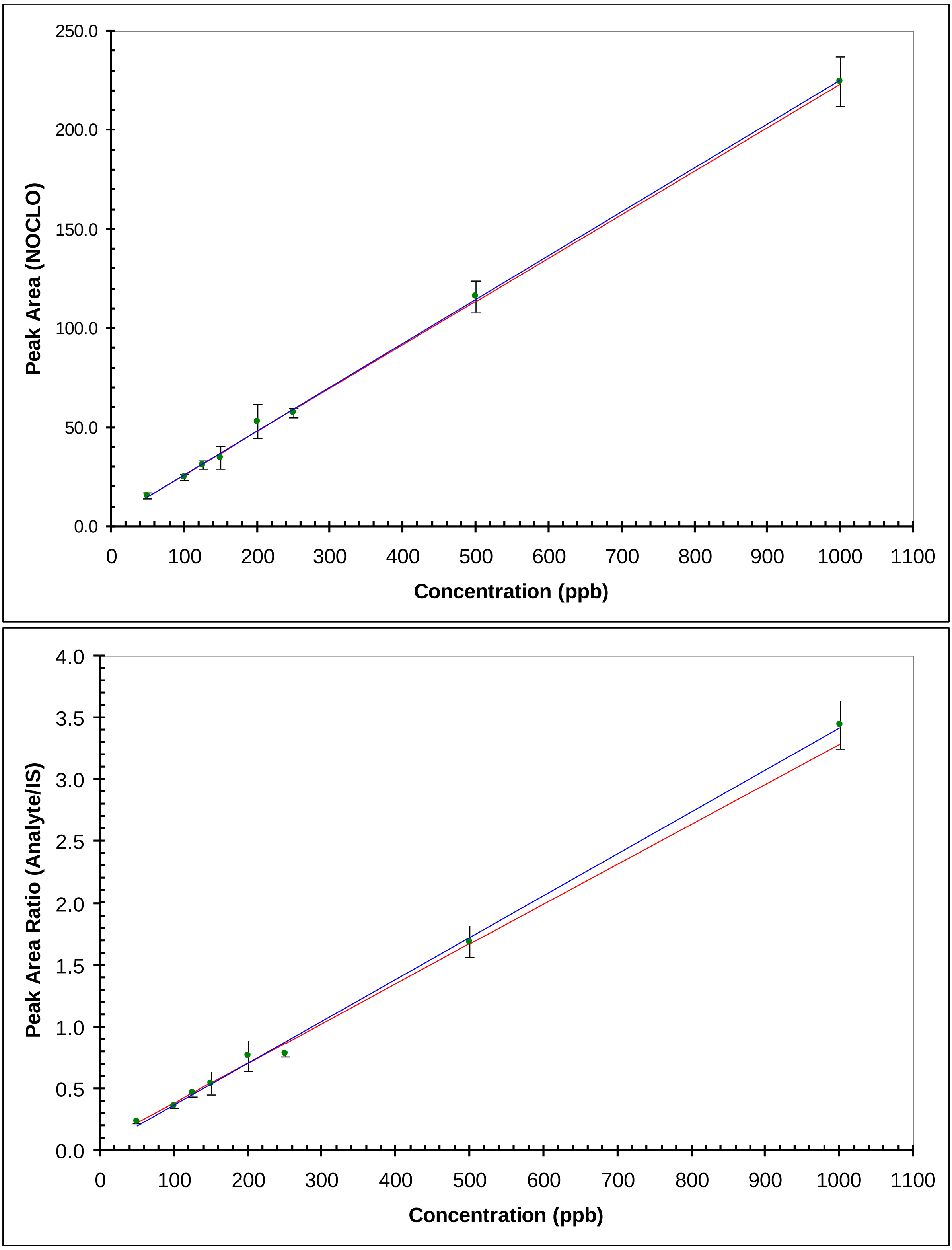

Figure S6. Calibrations for NOCLO in spiked plasma samples prepared through LLE in *n*-octanol, using either peak area (mAU*s) or analyte/IS peak area ratio (no units). Blue lines illustrate the linear regression model, while red lines represent the weighted $1/x^2$ linear regression model. Vertical bars indicate the normal variation intervals of the mean values obtained from six independent samples analyzed at each concentration level, expressed as +/- 2 x standard deviation.

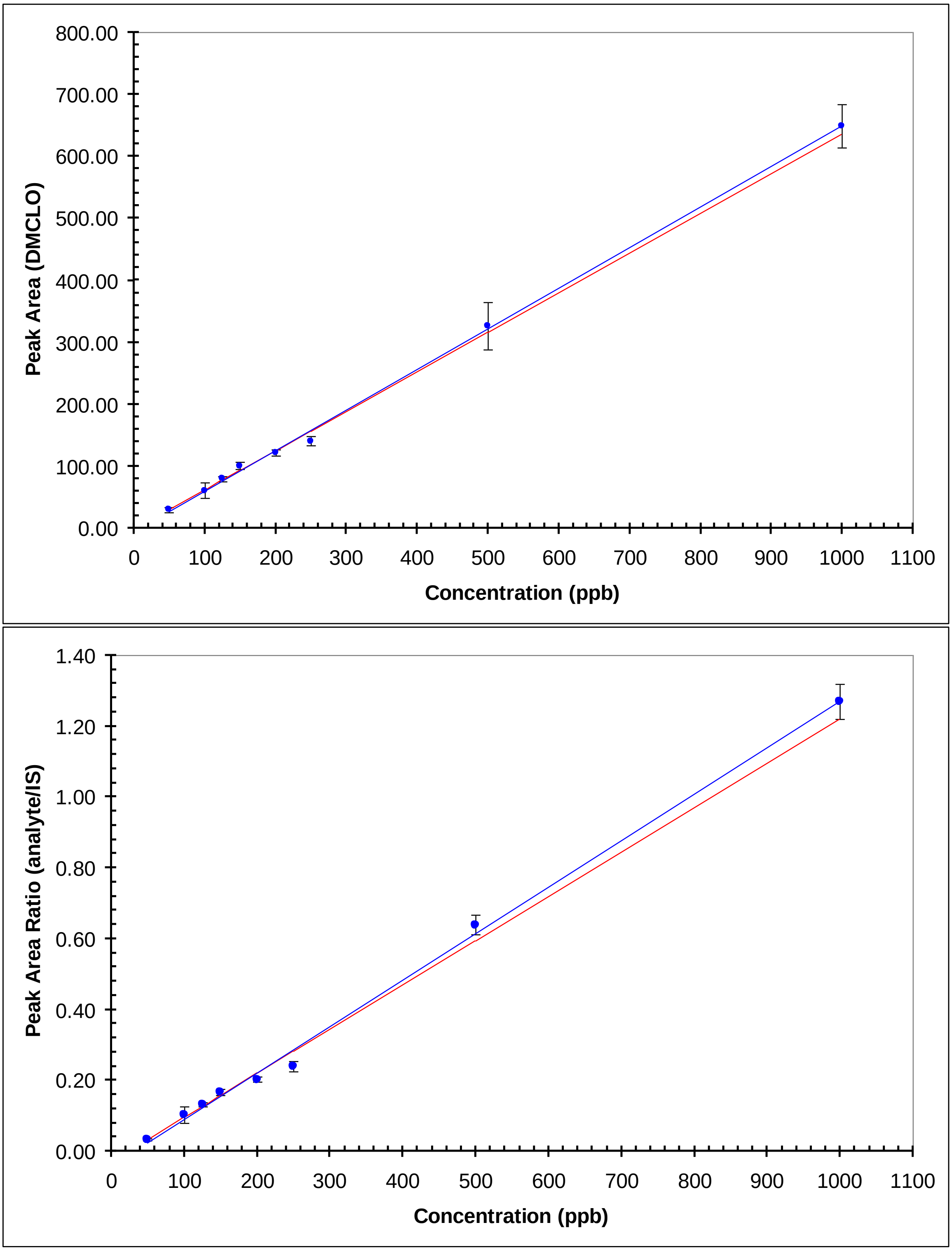

Figure S7. Calibrations for DMCLO in spiked urine samples prepared through LLE in *n*-octanol after alkalinization, using either peak area (mAU*s) or analyte/IS peak area ratio (no units). Blue lines illustrate the linear regression model, while red lines represent the weighted $1/x^2$ linear regression model. Vertical bars indicate the normal variation intervals of the mean values obtained from six independent samples analyzed at each concentration level, expressed as +/- 2 x standard deviation.

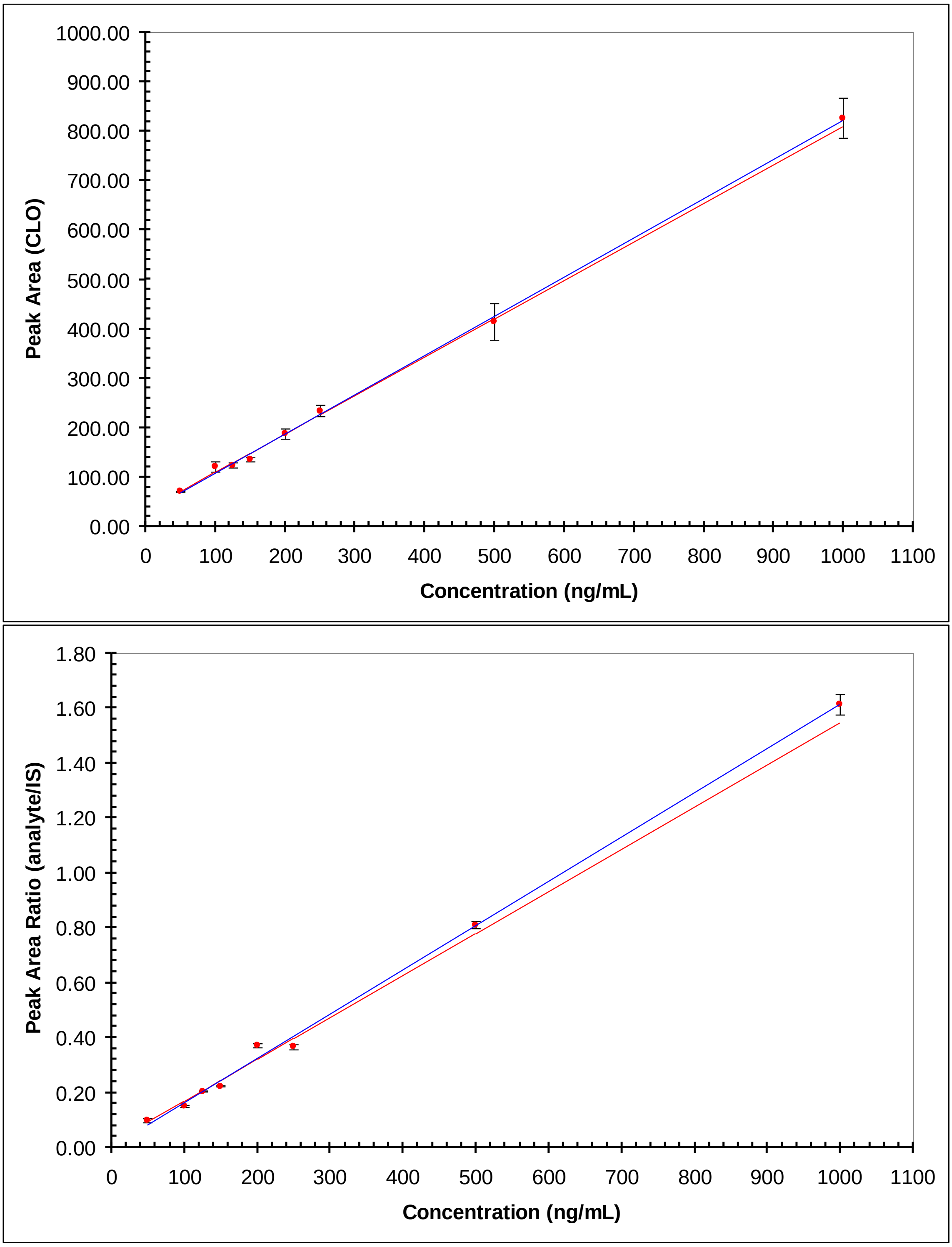

Figure 8. Calibrations for CLO in spiked urine samples prepared through LLE in *n*-octanol after alkalinization, using either peak area (mAU*s) or analyte/IS peak area ratio (no units). Blue lines illustrate the linear regression model, while red lines represent the weighted $1/x^2$ linear regression model. Vertical bars indicate the normal variation intervals of the mean values obtained from six independent samples analyzed at each concentration level, expressed as +/- 2 x standard deviation.

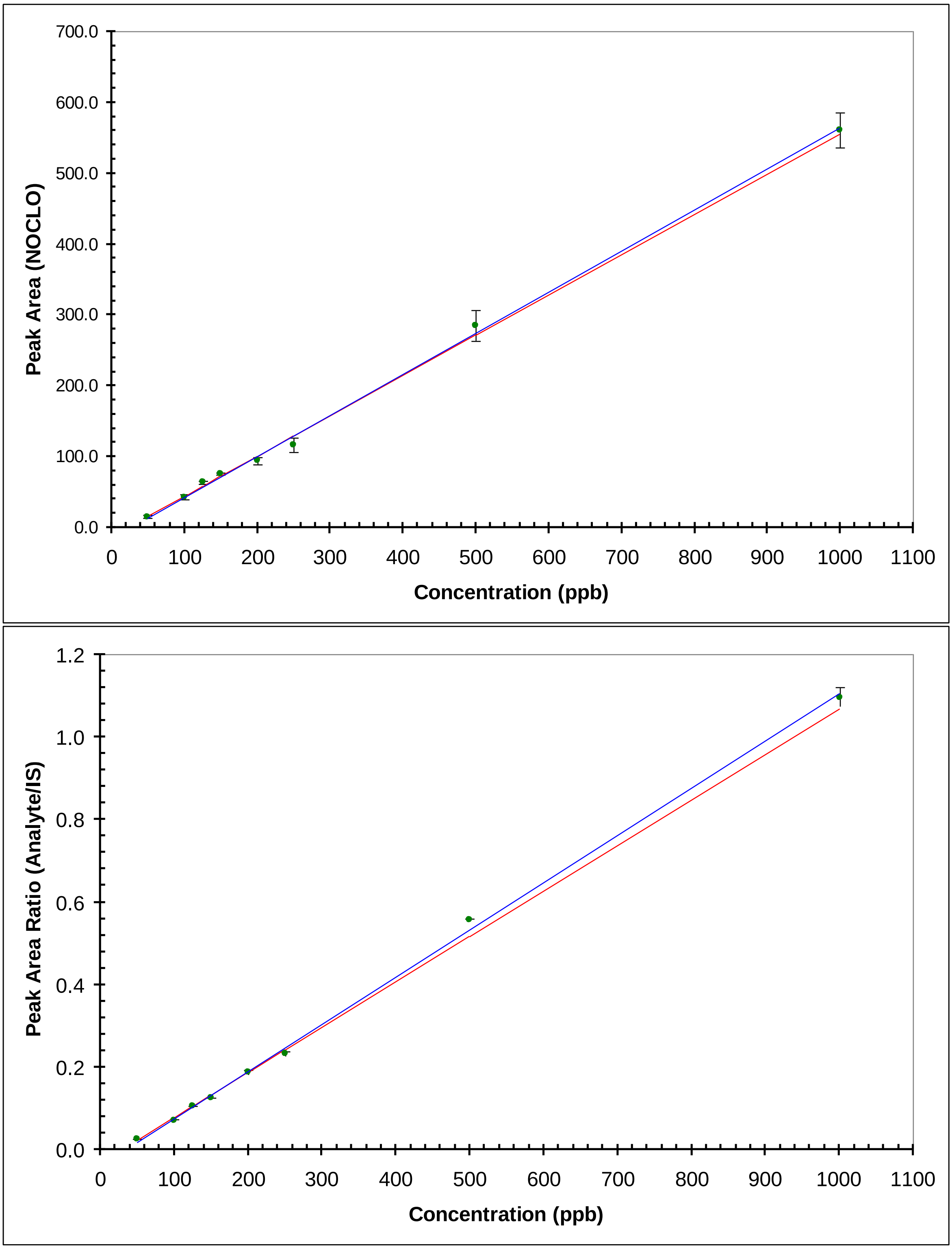

Figure S9. Calibrations for NOCLO in spiked urine samples prepared through LLE in *n*-octanol after alkalinization, using either peak area (mAU*s) or analyte/IS peak area ratio (no units). Blue lines illustrate the linear regression model, while red lines represent the weighted $1/x^2$ linear regression model. Vertical bars indicate the normal variation intervals of the mean values obtained from six independent samples analyzed at each concentration level, expressed as +/- 2 x standard deviation.

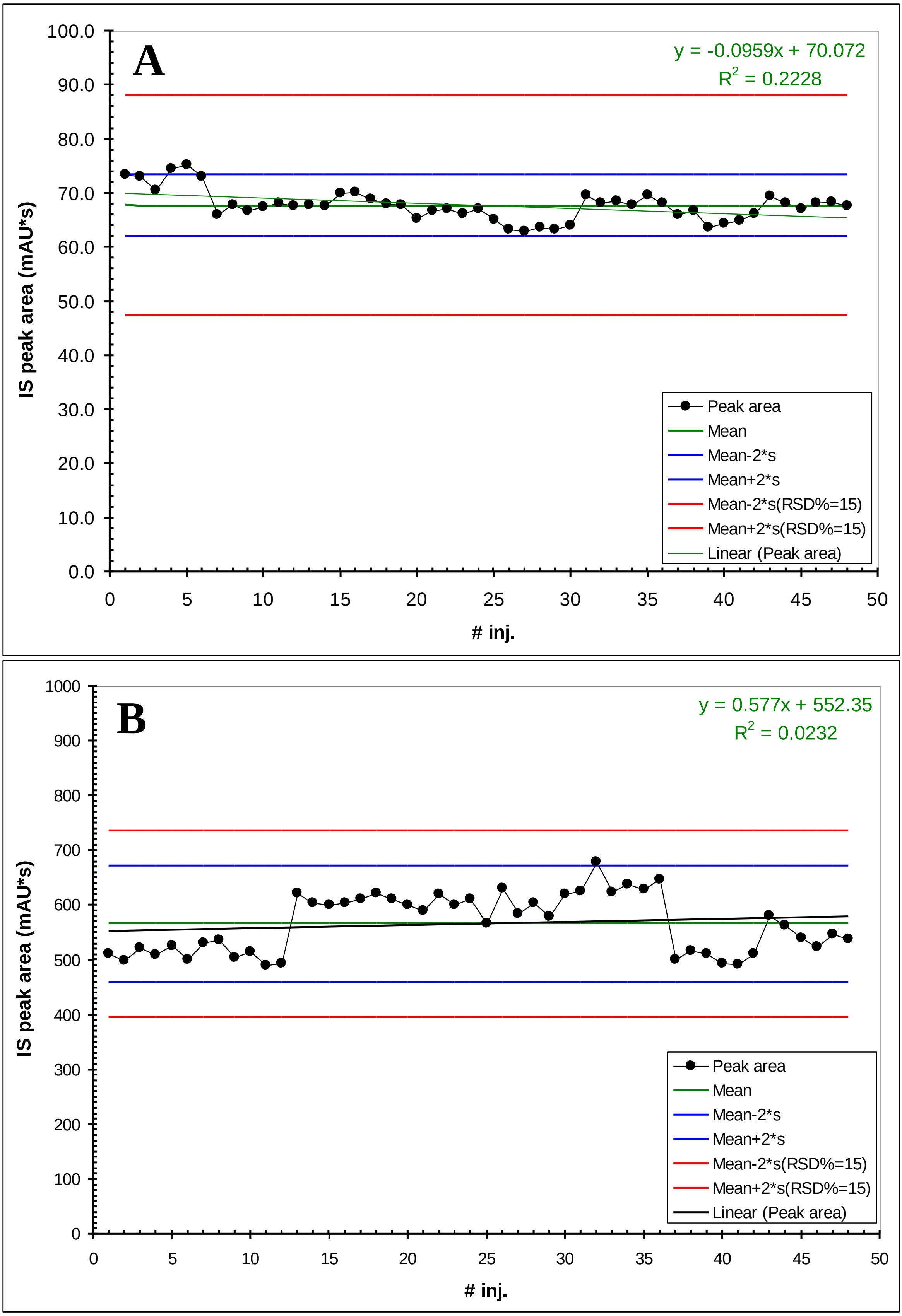

Figure S10. Variation of the IS peak areas values obtained during the response function study on analyzing plasma (A) and urine (B) matrices.

Table S1. Target analyte recovery on LLE extraction from human plasma and urine matrices in *n*-octanol.

Matrix	Analyte	Concentration ng/mL level	Mean peak areas in extracted spiked samples	Mean peak areas in post spiked samples (*n*-octanol)	Recovery (%)	Mean recovery (%)	Standard deviation	RSD %
Plasma	DMCLO	50	12.38	22.58	54.8	60.2	5.3242	8.8
		150	34.48	59.50	58.0			
		500	126.60	188.01	67.3			
		1000	242.37	398.31	60.8			
	CLO	50	15.65	26.13	59.9	59.9	3.7311	6.2
		150	39.82	71.00	56.1			
		500	134.03	206.33	65.0			
		1000	272.87	465.19	58.7			
	NOCLO	50	15.58	27.55	56.6	62.7	4.5002	7.2
		150	34.67	52.68	65.8			
		500	116.03	174.94	66.3			
		1000	224.65	361.82	62.1			
Urine	DMCLO	50	46.08	42.00	109.7	92.2	11.7481	12.7
		150	100.58	114.21	88.1			
		500	326.57	380.70	85.8			
		1000	648.85	761.40	85.2			
	CLO	50	69.73	54.60	127.7	109.9	12.6215	11.5
		150	135.27	122.94	110.0			
		500	414.35	409.80	101.1			
		1000	825.98	819.60	100.8			
	NOCLO	50	35.15	43.20	81.4	94.7	9.8438	10.4
		150	75.78	81.00	93.6			
		500	284.73	285.00	99.9			
		1000	561.02	540.00	103.9			

Table S2. Matrix effects on target analytes from LLE extraction of human plasma and urine matrices in *n*-octanol.

Matrix	Analyte	Concentration ng/mL level	Mean post-spike peak areas	Mean neat solution peak areas	Matrix effect	Mean	Standard deviation	RSD %
Plasma	DMCLO	50	22.58	19.95	1.1	1.1	0.04151	3.8
		150	59.50	55.58	1.1			
		500	188.01	179.27	1.0			
		1000	398.31	353.12	1.1			
	CLO	50	26.13	21.16	1.2	1.1	0.07333	6.4
		150	71.00	62.92	1.1			
		500	206.33	195.34	1.1			
		1000	465.19	410.98	1.1			
	NOCLO	50	27.55	20.52	1.3	1.2	0.10509	8.8
		150	52.68	45.60	1.2			
		500	174.94	158.46	1.1			
		1000	361.82	313.50	1.2			
Urine	DMCLO	50	42.00	42.00	1.00	1.0	0.020088	2.0
		150	114.21	117.00	0.98			
		500	380.70	377.40	1.01			
		1000	761.40	743.40	1.02			
	CLO	50	54.60	44.40	1.23	1.0	0.13761	13.4
		150	122.94	132.00	0.93			
		500	409.80	409.80	1.00			
		1000	819.60	862.20	0.95			
	NOCLO	50	43.20	43.20	1.00	0.9	0.08204	9.3
		150	81.00	96.00	0.84			
		500	285.00	333.60	0.85			
		1000	540.00	660.00	0.82			

Table S3. Long-term stability of stock solutions of DMCLO, CLO and NOCLO in DMSO (4 °C)

Concentration µg/mL / Amount loaded to column (ng)	Peak area units / ng injected			Mean	St. Dev.	RSD%
	**DMCLO**	**CLO**	**NOCLO**			
	Day 0	Day 62	Day 78			
5	2.70	2.53	2.44	2.56	0.1322	5.17
10	2.60	2.54	2.37	2.50	0.1173	4.69
12.5	2.27	2.27	2.07	2.20	0.1178	5.35
15	2.65	2.57	2.40	2.54	0.1290	5.08
20	2.66	2.61	2.40	2.56	0.1368	5.35
25	2.64	2.60	2.40	2.55	0.1332	5.23
50	2.63	2.62	2.39	2.55	0.1366	5.36
100	2.62	2.78	2.38	2.59	0.2060	7.94
Mean	2.60	2.57	2.36			
St. Dev.	0.1374	0.1422	0.1192			
RSD%	5.3	5.5	5.1			

Table S4. Stability data for target analytes and IS in the plasma matrix (recorded peak areas).

Stability	Analyte	DMCLO				CLO				NOCLO				IS				
	Conc.	50	150	500	1000	50	150	500	1000	50	150	500	1000	5 or 10 ppm				
Plasma at room temperature (25 oC)	0 h	12.4	34.5	126.6	242.4	15.7	39.8	134.0	272.9	15.6	34.7	116.0	224.7	36.6	33.3	32.6	34.1	for all values
	6 h	12.6	35.4	109.5	236.1	16.3	37.4	127.9	268.4	16.9	33.9	123.8	203.6	35.3	28.5	35.2	28.2	
	12 h	12.2	34.1	128.7	217.7	13.9	38.9	138.5	275.2	14.5	39.2	128.9	235.9	26.9	36.9	30.4	36.7	
	18 h	11.7	33.1	136.9	256.8	16.4	38.7	125.8	280.9	14.0	31.4	105.4	201.7	35.4	27.2	30.8	28.5	
	24 h	11.9	33.4	110.2	221.1	14.1	42.0	126.1	271.4	13.8	30.1	100.6	202.0	27.6	27.5	30.6	28.9	
	Mean	12.2	34.1	122.4	234.8	15.3	39.4	130.5	273.8	15.0	33.9	114.9	213.6	32.4	30.7	31.9	31.3	31.6
	St. Dev.	0.362	0.911	12.070	15.992	1.196	1.707	5.5832	4.6944	1.287	3.512	11.950	15.776	4.706	4.262	2.0363	3.8767	3.599
	RSD%	3.0	2.7	9.9	6.8	7.8	4.3	4.3	1.7	8.6	10.4	10.4	7.4	14.5	13.9	6.4	12.4	11.4
Processed plasma at room temperature (25 oC)	0 h	12.4	34.5	126.6	242.4	15.7	39.8	134.0	272.9	15.6	34.7	116.0	224.7	36.6	33.3	32.6	34.1	for all values
	12 h	12.6	34.1	126.9	236.8	16.1	39.3	133.8	269.1	15.4	30.1	118.7	220.3	27.8	32.1	27.4	34.8	
	24 h	12.4	34.4	102.2	205.6	15.3	42.9	116.6	251.4	14.4	30.4	92	184.5	29.9	28.7	28.8	28.9	
	36 h	12.2	33.1	100.6	204.0	14.2	42.3	114.0	248.2	14.6	35.1	90.1	176.5	35.8	26.4	32.1	28	
	48 h	12.1	33.2	100.5	204.1	14.3	42.5	116.6	251.6	15.3	30.3	90.8	182.7	29	27.5	27.1	28.3	
	Mean	12.3	33.9	111.4	218.6	15.1	41.4	123.0	258.6	15.1	32.1	101.5	197.7	31.8	29.6	29.6	30.8	30.5
	St. Dev.	0.193	0.661	14.065	19.290	0.835	1.671	10.016	11.432	0.523	2.535	14.506	22.834	4.086	2.982	2.6092	3.3346	3.173
	RSD%	1.6	2.0	12.6	8.8	5.5	4.0	8.1	4.4	3.5	7.9	14.3	11.5	12.8	10.1	8.8	10.8	10.4
Long term stability in frozen (-20 oC) plasma	24 h	12.9	33.8	120.4	220.1	13.7	45.4	136.4	275.1	15.6	38.3	125.2	232.8	66.7	62.9	74.5	70.2	for all values
	78 h	12.7	34.6	115.3	226.8	16.2	40.3	129.2	269.7	14.8	40.1	128.4	236.5	70.5	65.2	63.3	75.2	
	168 h	12.8	35.4	119.9	227.2	15.7	46.1	131.8	280.4	17	42.6	131.5	224.9	67.7	73.4	66.0	63.6	
	Mean	12.8	34.6	118.5	224.7	15.2	43.9	132.5	275.1	15.8	40.3	128.4	231.4	69.1	69.3	64.7	69.4	68.1
	St. Dev.	0.100	0.800	2.8113	3.9887	1.322	3.166	3.6460	5.3501	1.113	2.159	3.1501	5.9254	1.979	5.798	1.9092	8.2024	4.480
	RSD%	0.8	2.3	2.4	1.8	8.7	7.2	2.8	1.9	7.0	5.4	2.5	2.6	2.9	8.4	3.0	11.8	6.6
Unassisted freeze-thaw cycles	1 X	12.6	34.8	114.1	221.1	14.7	43.4	128	271.4	14.3	29.6	100.2	202	27	25.8	28.1	28.7	for all values
	2 X	12.6	34.4	110.4	223.3	15	42.5	123.8	268.6	15.9	30.8	99.9	198.8	28.6	27.3	26.7	26.7	
	3 X	12.5	33.4	114.9	227.9	15.1	41.6	130.6	276.1	16.4	29.8	104.4	204.1	27.5	26.5	27.3	26.6	
	Mean	12.6	34.2	113.1	224.1	14.9	42.5	127.5	272.0	15.5	30.1	101.5	201.6	27.7	26.5	27.4	27.3	27.2
	St. Dev.	0.057	0.721	2.4006	3.4698	0.208	0.900	3.4312	3.7899	1.097	0.642	2.5159	2.6690	0.818	0.750	0.7024	1.1846	0.877
	RSD%	0.5	2.1	2.1	1.5	1.4	2.1	2.7	1.4	7.1	2.1	2.5	1.3	3.0	2.8	2.6	4.3	3.2

Table S5. Stability data for target analytes and IS in the urine matrix (recorded peak areas).

Stability	Analyte	DMCLO				CLO				NOCLO				IS				
	Conc.	**50**	**150**	**500**	**1000**	**50**	**150**	**500**	**1000**	**50**	**150**	**500**	**1000**	**2 ppm**				
Urine at room temperature (25 °C)	0 h	29.6	100.6	326.6	648.9	69.7	135.3	414.4	826.0	25.2	75.8	284.7	561.0	511.7	605.7	504.7	549.1	for all values
	6 h	30.3	95.4	327.2	662.2	65.3	132.6	426.5	840.3	24.0	76.0	271.4	548.2	580.9	515.8	648.4	519.7	
	12 h	35.7	103.9	320.9	631.7	75.9	149.2	390.4	813.2	24.1	73.4	290.1	567.9	566.1	621.1	518.3	595.7	
	18 h	33.9	94.7	320.8	658.9	73.1	140.5	389.2	810.9	32.8	82.6	275.7	551.4	515.6	570.4	510.1	623.4	
	24 h	36.9	94.6	319.2	652.0	73.1	146.4	389.6	817.4	29.4	78.9	273.4	550.4	596.4	532.6	613.4	609.5	
	Mean	33.3	97.8	322.9	650.7	71.4	140.8	402.0	821.6	27.1	77.3	279.1	555.8	554.1	569.1	559.0	579.5	565.4
	St. Dev.	3.242	4.200	3.6752	11.891	4.062	7.068	17.356	11.953	3.879	3.530	8.0011	8.3651	38.50	45.33	66.988	43.560	46.78
	RSD%	9.7	4.3	1.1	1.8	5.7	5.0	4.3	1.5	14.3	4.6	2.9	1.5	6.9	8.0	12.0	7.5	8.3
Processed urine at room temperature (25 °C)	0 h	29.6	100.6	326.6	648.9	69.7	135.3	414.4	826.0	25.2	75.8	284.7	561.0	511.7	605.7	504.7	549.1	for all values
	12 h	27.3	92.7	299.5	597.5	70.1	138.7	409.8	825.2	24.8	74.5	271.3	554.9	534.5	521.1	548.2	610.4	
	24 h	26.8	63.1	200.8	397.0	67.6	137.8	385.9	831.9	37.0	74.1	269.1	561.0	582.7	520.8	597.9	606.7	
	36 h	20.8	52.4	146.2	280.4	70.6	135.2	382.1	833.6	30.7	78.6	278.1	559.7	583.4	580.2	587.7	565.8	
	48 h	22	44.6	138.6	268.5	73.1	138.6	387.0	829.1	34.4	75.6	270.6	560.9	584.2	525.6	601.2	602.7	
	Mean	25.3	70.7	222.3	438.5	70.2	137.1	395.8	829.2	30.4	75.7	274.8	559.5	559.3	550.7	567.9	586.9	566.2
	St. Dev.	3.726	24.75	86.733	176.88	1.972	1.751	15.027	3.640	5.445	1.762	6.556	2.632	34.01	39.68	41.187	27.695	35.82
	RSD%	14.7	35.0	39.0	40.3	2.8	1.3	3.8	0.4	17.9	2.3	2.4	0.5	6.1	7.2	7.3	4.7	6.3
Long-term stability in frozen (-20 °C) urine	24 h	36.1	105.4	336	700.5	58.7	167.4	554.6	924.7	30.6	80.5	278.2	522.6	498.6	625.3	590.1	621.9	for all values
	78 h	32.9	92.9	342.8	696.1	65.1	182.3	605.4	856.2	29.5	85.7	251.1	555.9	536.4	566.3	678.4	489.4	
	168 h	35.5	103.1	350.6	721.4	52.9	167.4	598.5	875.3	33.2	86.1	290.2	531.2	604.3	580.1	603.6	600.5	
	Mean	34.8	100.5	343.1	706.0	58.9	172.4	586.2	885.4	31.1	84.1	273.2	536.6	546.4	590.6	624.0	570.6	582.9
	St. Dev.	1.701	6.653	7.306	13.517	6.102	8.603	27.554	35.349	1.900	3.124	20.030	17.287	53.56	30.86	47.564	71.131	53.92
	RSD%	4.9	6.6	2.1	1.9	10.4	5.0	4.7	4.0	6.1	3.7	7.3	3.2	9.8	5.2	7.6	12.5	9.3
Unassisted freeze-thaw cycles	1 X	39.2	101	331	706.9	62	140	393.1	879.7	31.9	76.3	272.6	582.8	571.8	542.3	625.6	656.8	for all values
	2 X	40.3	99.5	345.3	645.5	69.7	141.1	407.3	799.6	34.7	77.1	278.3	535.6	602.5	542.2	640.2	602.5	
	3 X	36.9	101.7	335.6	645.8	64.4	145.7	408.5	815.9	30	76.9	279.9	540.7	527.3	544.8	646.2	608.4	
	Mean	38.8	100.7	337.3	666.1	65.4	142.3	403.0	831.7	32.2	76.8	276.9	553.0	567.2	543.1	637.3	622.6	592.6
	St. Dev.	1.735	1.124	7.300	35.363	3.940	3.024	8.566	42.332	2.364	0.416	3.837	25.905	37.81	1.473	10.595	29.793	45.57
	RSD%	4.5	1.1	2.2	5.3	6.0	2.1	2.1	5.1	7.3	0.5	1.4	4.7	6.7	0.3	1.7	4.8	7.7

Table S6. Exhaustive literature data search about cloazapine and related metabolites quatitative determination in biological matrices by means of chromatographic methods (focus was made on HPLC-DAD methods).

Reference	Analytes	Matrix	Chromato-graphic method	Column	Separation mechanism	Elution	Mobile phase additive	Detection	Sample preparation	LOQ
1	DMCLO, CLO, NOCLO	Dog plasma	HPLC	CN	RP	isocratic	Ammonium acetate pH=5	UV 254 nm	LLE - 2-butenol/cyclohexane 15/85 v/v Back-extraction in 0.1 M HCl	300 ng/mL
2	DMCLO, CLO	Human plasma	HPLC	ODS	RP	isocratic	KH_2PO_4 / $HClO_4$ / H_3PO_4	UV 230 nm	Plasma volume - 2 mL; LLE - alkalinization NaOH; hexane/*i*-amylalcohol 98.5/1.5 v/v; Back-extraction in 0.1M HCl	25 ng/mL
3	DMCLO, CLO	Human serum / plasma	Column switching	CN / ODS	RP	isocratic	Tetramethylene diamine/CH_3COOH	UV 254 nm	Direct injection (100 ◎L) in the first chromatographic dimension	20 ng/mL
4	DMCLO, CLO, NOCLO	Human plasma / red blood cells	HPLC	ODS	RP	isocratic	Phosphate buffer pH=7	UV 254 nm	LLE in ethylacetate; Back--extraction in 0.1 M HCl; Concentration by evaporation	20 - 30 ng/mL
5	DMCLO, CLO	Human plasma	HPLC	C8	IP	isocratic	Pic B5 / diethylamine	UV 245 nm	Plasma volume - 1 mL; LLE - alkalinization NaOH; hexane/*i*-amylalcohol 98.5/1.5 v/v; Back-extraction in 0.1M HCl	10 ng/mL
6	DMCLO, CLO	Rat serum	HPLC	ODS	RP	isocratic	CH_3COONa buffer pH=2.6	UV 230 nm	Alkalinization borate buffer pH=9; LLE in $CHCl_3$; evaporation to dryness	2.5 ng/mL
7	DMCLO, CLO, NOCLO	Human serum / rat plasma	HPLC	ODS	RP	isocratic	K_2HPO_4, pH=4	UV 254 nm	Automated SPE	15 ng/mL
8	DMCLO, CLO, NOCLO	Human serum / plasma	HPLC	ODS	RP	isocratic	Tetramethylethylene diamine / CH_3COOH pH=6.5	UV 254 nm	Automated SPE	60 ng/mL
9	DMCLO, CLO,	Human urine	UHPLC	ODS	RP	gradient	HCOOH 0.1%	UV 239 nm	Digitally controlled microextraction by	50 ng/mL

	NOCLO								packed sorbent (MEPS)	
10	DMCLO, CLO, NOCLO	Human plasma	HPLC	C8	RP	isocratic	34 mM phosphate buffer containing 0.3% triethylamine, pH=2	UV 254 nm	SPE Bondelut C1	20 ng/mL
11	DMCLO, CLO, NOCLO	Human plasma	HPLC	ODS	RP	isocratic	62.4 mM phosphate buffer containing 0.3% triethylamine at pH 4.5	UV 220 nm	LLE in ethyl acetate, *n*-hexane and isoamylalcohol (80:15:5, v/v/v); Concentration by evaporation	25 ng/mL
12	DMCLO, CLO, NOCLO	Human plasma	HPLC	C6	RP	isocratic	0.06 *M* phosphate buffer, pH 2.7 with 25% H_3PO_4	UV 254 nm	LLE *n*-hexane–isoamyl alcohol (75:25, v/v); Back-extraction in 0.1 *M* dibasic phosphate (pH 2.2 with 25% H_3PO_4)	5 ng/mL
13	DMCLO, CLO, NOCLO	Human plasma	HPLC	ODS	RP	isocratic	Dimethylamine 0.04%	UV 280 nm	LLE in ethyl acetate–*n*-hexane–isopropanol (16:3:1 (v:v:v)) twice; evaporation to dryness	6 ng/mL
14	DMCLO, CLO	Human serum / plasma	HPLC	C8	RP	isocratic	10 mM K_2HPO_4, pH=3.7	UV 220 nm	Automated SPE (ASPEC)	30 ng/mL
15	DMCLO, CLO	Human plasma	HPLC	ODS	RP	isocratic	Na_2HPO_4 / H_3PO_4 pH=4	UV 230 nm	1 mL plasma, LLE in *n*-hexane-isoamyl alcohol (98.5: 1.5, v/v); Back-extraction in 0.1M HCl; Re-extraction in *n*-hexane-isoamyl alcohol solution (98.5:1.5, v/v); evaporation to dryness	20 ng/mL
16	DMCLO, CLO	Human serum	HPLC	Bare Silica	NP	isocratic	CH_3COONH_4 buffer, pH=9.9	UV 261 nm	On-line SPE on CN cartridge	30 ng/mL
17	CLO	Human plasma	HPLC	ODS	RP	not reported	Not reported	UV spectral scanning	Multi-column (4) on-line	75 ng/mL
18	DMCLO, CLO, NOCLO	Human plasma	HPLC	ODS	RP	isocratic	50 mM NaH_2PO_4 pH=2.1	Coulometric	VAPD/VAPDmini	50 ng/mL
19	DMCLO, CLO, NOCLO	Blood (DBS)	HPLC	ODS	RP	isocratic	Phosphate buffer	Coulometric	Microextraction by packed column procedure	2.5 ng/mL

20	CLO	Human plasma	HPLC	ODS	RP	isocratic	0.25 mM CH_3COONH_4	Amperometric	alkalinization NaOH; LLE - hexane; evaporation to dryness	25 ng/mL
21	CLO	Whole blood	GC	OV-5	-	temperature gradient	He	MS (MID)	DLLME	15 ng/mL
22	CLO (among other 21 drugs)	Human plasma	HPLC	ODS	RP	gradient	10 mM CH_3COONH_4 (pH=8.0)	MS-MS	MASE (membrane assisted solvent extraction)	0.25 ng/mL
23	CLO	Human plasma	GC	OV-1	-	temperature gradient	He	Nitrogen Phosphorous Detector	SPME (on PDMS) - 30 min	100 ng/mL

1. Mosier, K.E.; Song, J.; McKay, G.; Hubbard, J.W.; Fang, J. Determination of clozapine, and its metabolites, *N*-desmethylclozapine and clozapine N-oxide in dog plasma using high-performance liquid chromatography, *J Chromatogr B*, **2003**, 783, 377–382.
2. Llerena, A.; Bereczi, R.; Norberto, M. J.; de la Rubia A. Determination of clozapine and its *N*-desmethyl metabolite by high-performance liquid chromatography with ultraviolet detection, *J Chromatogr B*, **2001**, 755, 349–354.
3. Weigmann, H.; Hartter, S.; Maehrlein, S.; Kiefer, W.; Kramer, G.; Dannhardt, G.; Hiemke, C. Simultaneous determination of olanzapine, clozapine and demethylated metabolites in serum by on-line column-switching high-performance liquid chromatography, *J Chromatogr B*, **2001**, 759, 63–71.
4. Guitton, C.J.; Kinowski, M.; Aznar, R.; Bressolle, F. Determination of clozapine and its major metabolites in human plasma and red cells by high-performance liquid chromatography with ultraviolet absorbance detector, *J Chromatogr B*, **1997**, 690, 211-222.
5. Edno, L.; Combourieu, I.; Cazenave, M.; Tignol, J. Assay for quantitation of clozapine and its metabolite *N*-desmethylclozapine in human plasma by high-performance liquid chromatography with ultraviolet detection, *J Pharm Biomed Anal*, **1997**, 16, 311-318.
6. Ma, F.; Lau, C.E. Determination of clozapine and its metabolite, N-desmethylclozapine, in serum microsamples by high-performance liquid chromatography and its application to pharmacokinetics in rats, *J Chromatogr B*, **1998**, 712, 193-198.
7. Fadiran, E.O.; Leslie, J.; Fossler, M.; Young, D. Determination of clozapine and its major metabolites in human serum and rat plasma by liquid chromatography using solid-phase extraction and ultraviolet detection, *J Pharm Biomed Anal*, **1995**, 13, 185-190.
8. Weigmann, H.; Hiemke, C. Determination of clozapine and its major metabolites in human serum using automated solid-phase extraction and subsequent isocratic high-performance liquid chromatography with ultraviolet detection, *J Chromatogr*, **1992**, 583, 209-216.
9. Gonçalves, J.L.; Alves, V.L.; Conceição, C.J.F.; Teixeira, H.M.; Camara, J.S. Development of MEPS–UHPLC/PDA methodology for the quantification of clozapine, risperidone

and their major active metabolites in human urine, Microchem *J*, **2015,** 123, 90-98.

10. Mercolini, L.; Bugamelli, F.; Kenndler, E.; Boncompagni, G.; Franchini, L.; Raggi, M.A. Simultaneous determination of the antipsychotic drugs levomepromazine and clozapine and their main metabolites in human plasma by a HPLC-UV method with solid-phase extraction, *J Chromatogr B*, **2007**, 846, 273-280.
11. Dural, E.; Mergen, G.; Soylemezoglu, T. Determination and quantitation of clozapine and its metabolites in plasma by HPLC, *Toxicol Lett*, **2009**, 189S, S57–S273.
12. Avenoso, A.; Facciola, G.; Campo, G.M.; Fazio, A.; Spina, E. Determination of clozapine, desmethylclozapine and clozapine *N*-oxide in human plasma by reversed-phase high-performance liquid chromatography with ultraviolet detection, *J Chromatogr B*, **1998**, 714, 299-308.
13. Shen, Y.L.; Wu, H.L.; Ko, W.K.; Wu, S.M. Simultaneous determination of clozapine, clozapine *N*-oxide, *N*-desmethylclozapine, risperidone, and 9-hydroxyrisperidone in plasma by high performance liquid chromatography with ultraviolet detection, *Anal Chim Acta* **2002**, 460, 201–208.
14. Akerman, K.K. Analysis of clozapine and norclozapine by high-performance liquid chromatography, *J Chromatogr B*, 1997, 696, 253-259.
15. Chung, M.-C.; Lin, S.-K.; Chang, W.-H.; Jann, M.W. Determination of clozapine and desmethylclozapine in human plasma by high-performance liquid chromatography with ultraviolet detection, *J Chromatogr*, **1993**, 613, 168-173.
16. Olesen, O.V.; Poulsen, B. On-line fully automated determination of clozapine and desmethylclozapine in human serum by solid-phase extraction on exchangeable cartridges and liquid chromatography using a methanol buffer mobile phase on unmodified silica, *J Chromatogr*, **1993**, 622, 39-46.
17. Wong, J.O.-Y.; Leung, S.-P.; Mak, T.; Man-kin Ng, R.; Chan, K.-T.; Cheung, H.H.-K.; Choi, W.-K.; Lai, J.; Tsang, A.W.-K. Plasma clozapine levels and clinical response in treatment-refractory Chinese schizophrenic patients, *Progress in Neuro-Psychopharmacol & Biol Psychiatry* **2006**, 30, 251 – 264.
18. Nakahara, T.; Otani, N.; Ueno, T.; Hashimoto, K. Development of a hematocrit-insensitive device to collect accurate volumes of dried blood spots without specialized skills for measuring clozapine and its metabolites as model analytes, *J Chromatogr B*, **2018**, 1087-1088, 70-79.
19. Saracino, M.A.; Lazzara, G.; Prugnoli, B.; Raggi, M.A. Rapid assays of clozapine and its metabolites in dried blood spots by liquid chromatography and microextraction by packed sorbent procedure, *J Chromatogr A*, **2011,** 1218, 2153-2159.
20. Humpel, C.; Haring, C.; Saria, A. Rapid and sensitive determination of clozapine in human plasma using high-performance liquid chromatography and amperometric detection, *J Chromatogr Biomed Appl*, **1989**, 491, 235-239.
21. Chen, X.; Zheng, S.; Le, J.; Qian, Z.; Zhang, R.; Hong, Z.; Chai, Y. Ultrasound-assisted low-density solvent dispersive liquid–liquid microextraction for the simultaneous determination of 12 new antidepressants and 2 antipsychotics in whole blood by gas chromatography–mass spectrometry, *J Pharm Biomed Anal*, **2017**, 142, 19-27.
22. Vega, A.J.; Jinadasa, K.K.; Thilini Madurangika Jayasinghe, G.D.; Alvarez-Freire, I.; Bermejo, A.M.; Bermejo-Barrera, P.; Moreda-Pineiro, A. Ultrasound assisted membrane-assisted solvent extraction for the simultaneous assessment of some drugs involved in drug-facilitated sexual assaults by liquid chromatography-tandem mass spectrometry, *J Chromatogr A*, **2023**, 1706, 464284.
23. Ulrich, S.; Kruggel, S.; Weigmann, H.; Hiemke, C. Fishing for a drug: solid-phase microextraction for the assay of clozapine in human plasma, *J Chromatogr B*, **1999**, 731, 231–240.

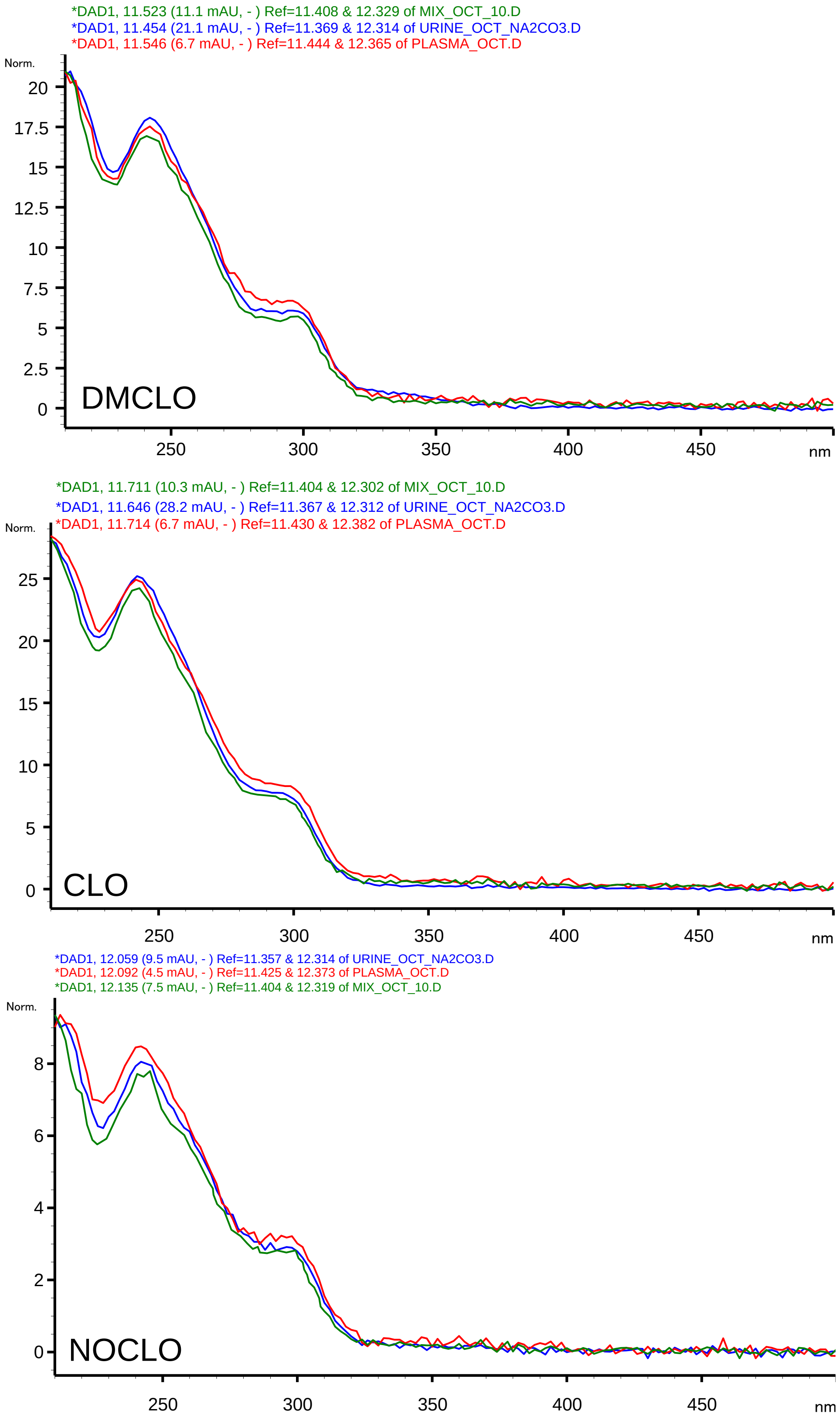

Figure S11. Overlaid UV spectra of the target analytes at the peak apex at LOQ level spiked biological matrices (urine - blue trace; plasma - red trace) compared to the spectra of the analytes in the chromatogram of a neat *n*-octanol solution (green trace).

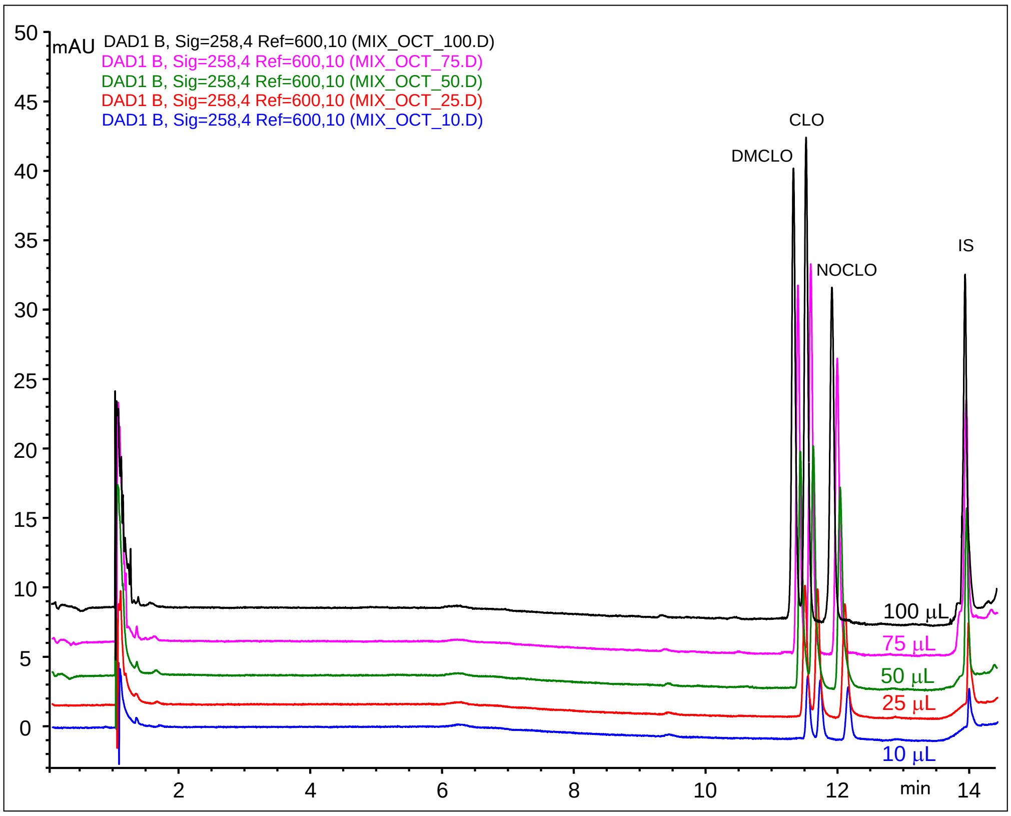

Figure S12. Overlaid chromatograms of increased injection volumes (10 - 100 µL) of the analytes in *n*-octanol.

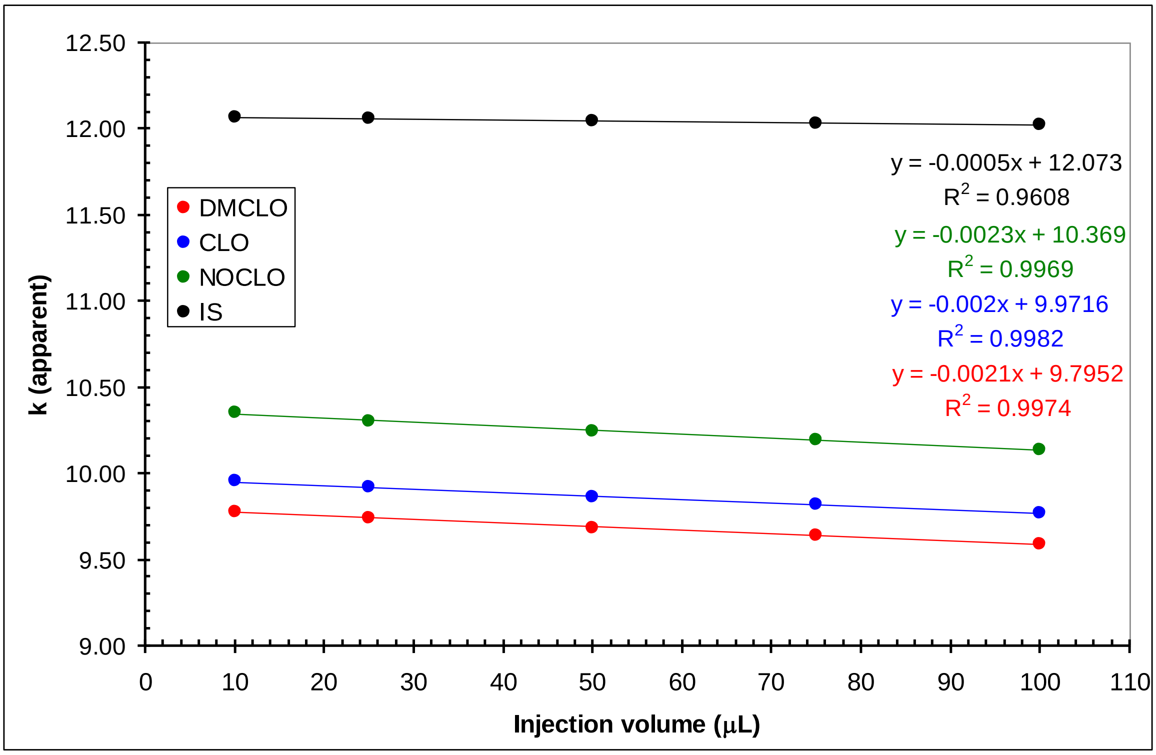

Figure S13. Apparent retention factor decrease for the analytes with *n*-octanol injection volume increase.

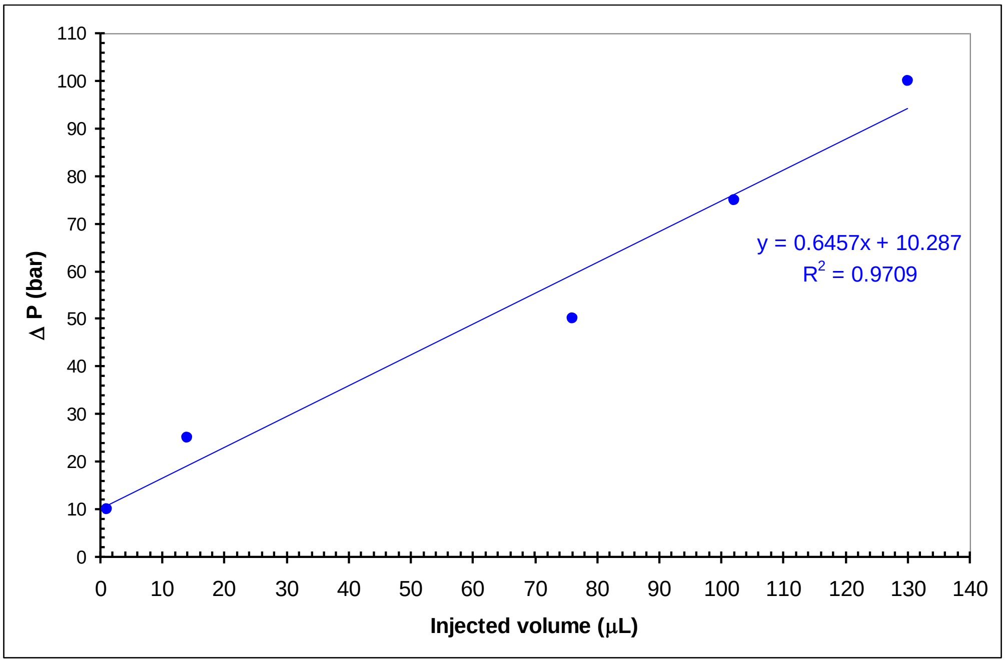

Figure S14. Variation of column pressure drop with increased injection volumes of solutions/extracts in *n*-octanol, registered during the sample plug transfer into the column.

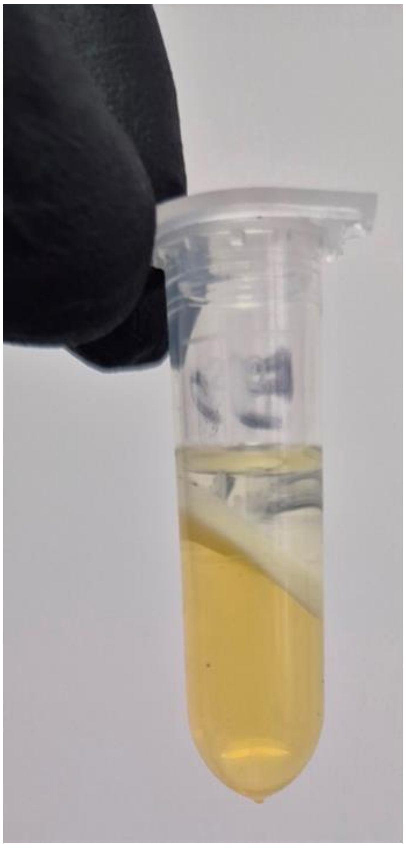

Figure S15. Layer separation after centrifugation of plasma samples extracted with *n*-octanol. Attention should be paid to the intermediate layer separating plasma from *n*-octanol extract, which makes collection easier, but incorporates some of the extracting solvent. The total *n*-octanol volume recovered after extraction can be fairy approximated to 0.35 mL from 0.5 mL used in the process (around 70%).
